# Genomic Variation and Its Impact on Gene Expression in *Drosophila melanogaster*

**DOI:** 10.1371/journal.pgen.1003055

**Published:** 2012-11-15

**Authors:** Andreas Massouras, Sebastian M. Waszak, Monica Albarca-Aguilera, Korneel Hens, Wiebke Holcombe, Julien F. Ayroles, Emmanouil T. Dermitzakis, Eric A. Stone, Jeffrey D. Jensen, Trudy F. C. Mackay, Bart Deplancke

**Affiliations:** 1 Laboratory of Systems Biology and Genetics, Institute of Bioengineering, School of Life Sciences, École Polytechnique Fédérale de Lausanne (EPFL), Lausanne, Switzerland; 2 Department of Organismic and Evolutionary Biology, Harvard University, Cambridge, Massachusetts, United States of America; 3 Department of Genetic Medicine and Development, University of Geneva Medical School, Geneva, Switzerland; 4 Department of Genetics, North Carolina State University, Raleigh, North Carolina, United States of America; 5 Institute of Bioengineering, School of Life Sciences, École Polytechnique Fédérale de Lausanne (EPFL), Lausanne, Switzerland; National Institutes of Health, United States of America

## Abstract

Understanding the relationship between genetic and phenotypic variation is one of the great outstanding challenges in biology. To meet this challenge, comprehensive genomic variation maps of human as well as of model organism populations are required. Here, we present a nucleotide resolution catalog of single-nucleotide, multi-nucleotide, and structural variants in 39 *Drosophila melanogaster* Genetic Reference Panel inbred lines. Using an integrative, local assembly-based approach for variant discovery, we identify more than 3.6 million distinct variants, among which were more than 800,000 unique insertions, deletions (indels), and complex variants (1 to 6,000 bp). While the SNP density is higher near other variants, we find that variants themselves are not mutagenic, nor are regions with high variant density particularly mutation-prone. Rather, our data suggest that the elevated SNP density around variants is mainly due to population-level processes. We also provide insights into the regulatory architecture of gene expression variation in adult flies by mapping *cis-*expression quantitative trait loci (*cis-*eQTLs) for more than 2,000 genes. Indels comprise around 10% of all *cis*-eQTLs and show larger effects than SNP *cis-*eQTLs. In addition, we identified two-fold more gene associations in males as compared to females and found that most *cis-*eQTLs are sex-specific, revealing a partial decoupling of the genomic architecture between the sexes as well as the importance of genetic factors in mediating sex-biased gene expression. Finally, we performed RNA-seq-based allelic expression imbalance analyses in the offspring of crosses between sequenced lines, which revealed that the majority of strong *cis*-eQTLs can be validated in heterozygous individuals.

## Author Summary

One of the principal challenges in current biology is to understand the relationship between genetic and phenotypic variation. The increasing availability of genomic variation maps of human as well as of model organism populations (mouse and *Arabidopsis*) constitutes an important step towards meeting this challenge. However, despite its excellent track record as a premier model to understand genome function, no genome-wide variation data beyond single-nucleotide variants and microsatellites are currently available for *D. melanogaster*. Here, we present a comprehensive, nucleotide-resolution catalogue of variants of various types (single-nucleotide, multi-nucleotide, and structural variants) for 39 wild-derived inbred *D. melanogaster* lines based on high-throughput sequencing. This catalogue confirms that non–SNP variants account for more than half of genomic variation, allowing us to provide new insights into the non-random distribution of variants in the *Drosophila* genome. We further present genome-wide *cis-*associations with gene expression based on whole adult fly microarray data, revealing significant associations for about 2,000 genes. Most associations are sex-specific, providing evidence for a decoupling of the genomic, regulatory architecture between males and females.

## Introduction

An important challenge in biology is to elucidate the relationship between genetic and phenotypic variation [1]. The increasing availability of comprehensive genome sequences of both human [2], [3] and model organism populations [4], [5] constitutes an important step towards meeting this challenge. The *Drosophila* Genetic Reference Panel (DGRP) is an example of such a recently emerging population resource, consisting of 192 sequenced wild-derived inbred *Drosophila melanogaster* lines [6], [7]. *Drosophila* is a premier model organism to understand genome function given the availability of powerful and cost-effective genetic tools and resources [8]–[10]. In addition, its genome is small, but highly polymorphic [7], [11]–[15], which has already proven helpful in studying the molecular basis of morphological evolution [16], [17]. Moreover, linkage disequilibrium (LD) decays quickly across the genome [7], [18], which is favorable to elucidating the relationship between genotypic and phenotypic variation at high resolution. This requires that we genotype all classes of segregating variants (*i.e.*, insertions, deletions, and structural variants) and not only the commonly studied single nucleotide polymorphisms (SNPs) to investigate their effect on phenotypes. Here, we use an integrative approach to derive and characterize a genome-wide, nucleotide-resolution catalog of variants including SNPs, insertions, deletions, complex substitutions, and structural variants in 39 DGRP lines. We then investigate the impact of naturally occurring genetic variation on adult gene expression, revealing novel insights into the regulatory architecture of gene expression variation in *Drosophila*.

## Results

### Variant discovery

**Figure 1:**
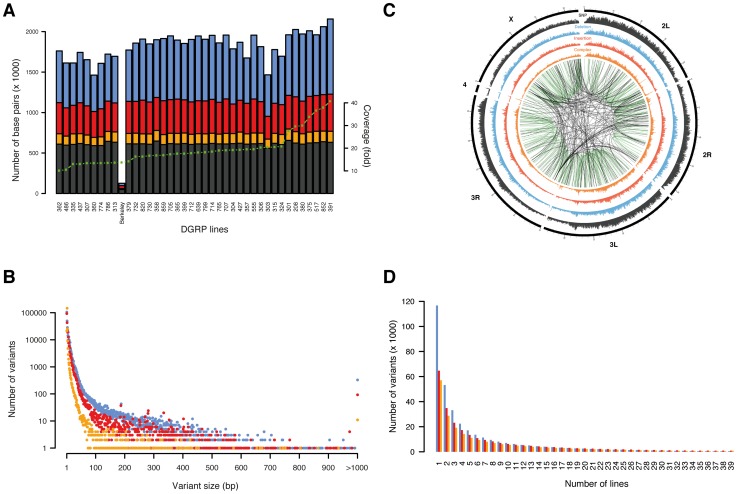
Overview of variants. SNPs are shown in black/grey, insertions in red, deletions in blue, and complex variants in orange. (A) Number of base pairs affected by variants discovered per line, with lines ordered by depth of coverage (green dotted line). The line “Berkeley” is the reference line. (B) Number of unique variants by size (note that variants longer than 1,000 bp are grouped in a single x-coordinate). (C) Representation of variant density (0–10 SNPs/kb, 0–5 indels/kb, 0–5 complex variants/kb) across the euchromatic genome (concentric circles) in 50 kb bins. Large variants (>100 bp) mapping against a close homologous sequence (>90% sequence identify) are linked in the center with green lines representing intra-chromosomal- and black lines inter-chromosomal duplications. (D) Number of unique variants by number of lines.

We generated a catalog of sequence variants using whole-genome Illumina next-generation sequencing data from 39 inbred lines from the *Drosophila* Genetic Reference Panel (DGRP) for which gene expression data are available for young adult flies [6]. First, we used PrInSeS-G [19], which uses *de novo* local assembly to generate a preliminary list of variant calls for each DGRP line; this tool works by first detecting regions with a fluctuation in sequencing coverage, then using a short fragment from the reference sequence as a seed to build a contig by extending it using the reads; the contig extending stops when another short fragment from the reference sequence is encountered. We then re-aligned each resulting genome against the reference genome to present variants in a coherent way among lines and to reduce variant fragmentation. Finally, we developed and used a genotyping algorithm, which uses the combined variant call set for all DGRP lines to improve variant discovery. This is particularly useful for genomes with low read coverage (Figure S1). This strategy allowed us to identify more than 3.6 million unique sequence variants across all 39 DGRP lines including 2.8 million SNPs, 0.6 million indels, and 0.2 million complex sequence variants (Figure 1A, Table 1). To validate our variant calls, we used five distinct approaches. First, we compared our variant catalogue to indel data from the FLYSNPdb database [20]. We validated 713 deletions and 923 insertions on the breakpoint and complete sequence level, thus covering in total 45% of the FLYSNPdb content. Second, we compared our SNP calls with those published by Mackay *et al*. [7], and found that 94% of calls made in that study match (Table S1). Third, we used whole genome Roche-454 reads available for the same lines to validate our SNP calls, resulting in confirmation of 98.7% of SNPs compared (Table S2). Fourth, we sequenced mRNA from three DGRP lines (see also below) and differentially aligned the reads to their respective transcriptomes and the reference transcriptome in order to validate variants in coding sequence. Thus, we examined, on average, 147,453 SNPs and 12,084 non-SNPs per line that were covered by mRNA sequencing reads, confirming on average 98.9% of all variant types within coding sequence for these lines (Table S3). The false discovery rate (FDR) is 0.5% for SNPs and 1.5% for indels and complex variants. Moreover, the FDR remains stable for indels up to 30 bp in size but increases to 8.9% for larger indels. Note that some variants could not be reliably assigned to the true or false positive categories due to low coverage. Finally, we validated 92 large indels (200 bp to 5.6 kb) in five DGRP lines using PCR (true positives 77.9%, false positives 7.2%, variant size different from predicted 14.9%, Table S4). Moreover, Sanger sequencing of 10 randomly selected large indels validated by PCR revealed a high breakpoint accuracy, whereby breakpoints of 9 out of 10 indels were perfectly reconstructed consistent with results based on query of smaller indels in the FLYSNPdb database. Together, these results indicate that our variant catalogue is of similar quality as variant data produced for other organisms using high-throughput sequencing since an overall false discovery rate of less than 10% and an overall accuracy of ∼95% or higher for all variant types (*i.e.*, SNPs, indels and complex variants) is in line with numbers reported by these other studies [4], [21]–[23].

**Table 1:** Type classification of variants.

Categories				Conditions	Total number (unique in 39 lines)	Average number per line	Mean total *nd* per line	Mean total *ni* per line	Mean *nd* per variant	Mean *ni* per variant
Variant				**nd > 0 or ni > 0**	3,667,449	761,417	1,438,915	1,134,852	1.9	1.5
	SNP - Single Nucleotide Polymorphism			**nd = ni = 1**	2,829,654	615,258	615,258	615,258	1	1
	Indel - Insertion/Deletion			**nd = 0 or ni = 0**	617,856	104,681	697,031	393,905	6.7	3.8
		Insertion		**nd = 0**	263,416	52,227	-	393,905	-	7.5
			Homopolymer	repeat size = 1, resulting homopolymer size ≥ 3	92,967	16,436	-	22,398	-	1.4
			Microsatellite	2 ≤ repeat size ≤ 4, resulting number of repeats ≥ 4	15,603	2,346	-	9,336	-	4.0
			Tandem Repeat	repeat size > 4, resulting number of repeats ≥ 2	11,234	2,126	-	15,978	-	7.5
			Segmental Duplication / Copy Number Variant	ni ≥ 100 blat 90% match, 98% identity, bitscore ≥ 300	315	30	-	12,429	-	414
		Deletion		**ni = 0**	354,440	52,454	697,031	-	13.3	-
			Homopolymer	repeat size = 1, reference homopolymer size ≥ 3	105,675	17,803	25,805	-	1.4	-
			Microsatellite	2 ≤ repeat size ≤ 4, reference number of repeats ≥ 4	17,661	2,256	9,105	-	4.0	-
			Tandem Repeat	repeat size > 4, reference number of repeats ≥ 2	14,309	1,688	12,867	-	7.6	-
			Segmental Duplication / Copy Number Variant	nd ≥ 100 blat 90% match, 98% identity, bitscore ≥ 300	587	213	295,094	-	1,385	-
	Complex			**ni > 0 and nd > 0**	219,939	41,478	126,626	125,690	3.1	3.0
		MNS - Multiple Nucleotide Substitution		nd = ni > 1	161,432	30,973	69,799	69,799	2.3	2.3
			Inversion	nd = ni ≥ 4, sequence replaced by its reverse complement	511	98	447	447	4.6	4.6

### Indels and complex variants

Among all lines, we identified a SNP every 43 bp and an indel or complex variant every 144 bp, together contributing a genetic marker every 33 bp on average. These findings illustrate a remarkably high density of molecular polymorphisms in *Drosophila* consistently greater than in humans [3], [24] and mice [4]. Deletions and complex variants affect in total 4.2% (5.0 Mb) of the reference euchromatic genome while insertions add 2.1 Mb (of which 0.5 Mb are in insertions ≥100 bp not found with similarity of 90% or above elsewhere in the reference genome). Non-SNP variants are thus a substantial source of genomic variation in *Drosophila*. Indel size ranges from 1 to 6,082 bp (Table 1, Figure 1B). Single base pair indels are abundant (32%) and small indels (2–10 bp) represent 50% of the indel repertoire. 6,419 structural variants (≥100 bp) represent 1% of all indels with a median size of 208 bp and encompass ∼2.5 Mb of sequence in total. 850 structural variants are homologous (≥90% sequence similarity) to another part of the assembled reference genome, representing either “young” variants or polymorphic forms of segmental duplications (*i.e.*, copy number variants) [25]. Most (70%) of those structural variants correspond to a duplicon on the same chromosome, indicating that intra-chromosomal duplication events occur more frequently (Figure 1C). These observations support a recently proposed theoretical model governing the formation of segmental duplications in *D. melanogaster*, whereby, after a double strand break, a search for an ectopic homologous region which is preferentially located within the same chromosomal region triggers the repair mechanism [26].

The majority (73%) of complex variants are balanced multi-nucleotide substitutions and most (61%) constitute di-nucleotide substitutions. Balanced multi-nucleotide substitutions may arise as single nucleotide substitutions that happen to occur in adjacent positions or as multi-nucleotide mutational events [27]. Using simulation within all 1,000 bp genomic windows in all 39 lines, we find that there are on average 3.3 times more di- and 16.3 times more tri-nucleotide substitutions than the number of single nucleotide substitutions expected to be adjacent by chance, indicating that complex mutational events constribute substantially to balanced multi-nucleotide substitutions.

The allele frequency spectrum of indels and complex variants decays steeply, with 28.5% of all indels and complex variants present in only one line (Figure 1D). Comparison to neutral spectra (Figure S2) revealed an excess of low frequency alleles for deletions, consistent with previous results suggesting that more of these variants are under purifying selection [13]. We also observed a depletion of high frequency variants among indels and complex variants in protein-coding regions, splice junctions, and UTR sequences (Figure S3), again suggesting the action of pervasive purifying selection. In contrast, we found 452 deletions, 873 insertions, and 824 complex variants present among all 39 lines (Table S5). To evaluate whether these variants represent rare alleles in the reference genome, population-specific variants, or artifacts in our variant calling workflow, we focused on all 30 such insertions and deletions that were present in protein-coding regions. We found that 77% of these variants exactly recapitulate an ancestral allele (*D. simulans*, *D. yakuba*, or *D. ananassae*) and 90% when allowing at most one mismatch between the indel and the ancestral allele (Table S6). Therefore, these variants predominantly constitute rare alleles in the reference genome. The photoreceptor gene *Rh6* is an interesting example: evolutionary conservation analysis around the two observed 17 bp and 2 bp insertions in its coding sequence revealed that both insertions perfectly match the ancestral allele of seven out of 11 *Drosophila* species (Figure S4). Moreover, the resulting gene model supports an *Rh6* cDNA clone of the OregonR/white strain, thus revealing that the reference genome harbors a rare 19 bp deletion, which truncates the gene.

**Figure 2:**
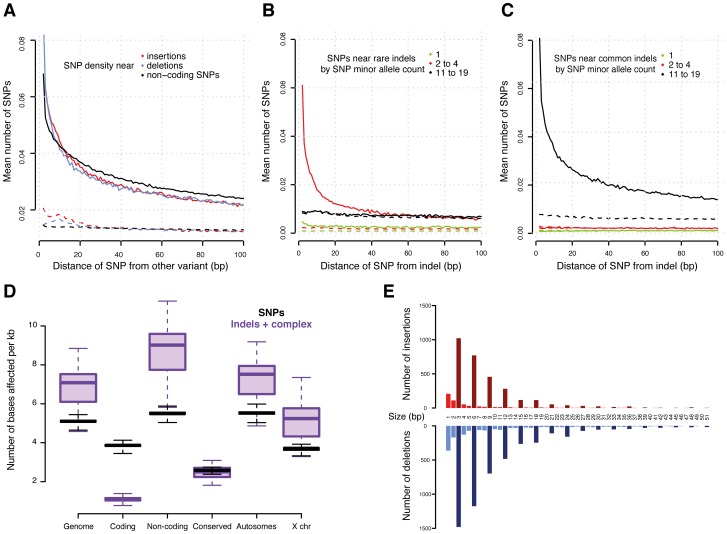
Variants in genomic context. (A) Density of SNPs around variant breakpoints by variant type. The dashed lines show the SNP density at the same loci but in DGRP lines that do not have the variant. (B) and (C) Density of SNPs near indels with minor allele count 2 to 4 (B) and 11 to 19 (C). The dashed lines show the SNP density at the same locus for DGRP lines without the indel. If indels were mutagenic, one would expect enrichment for low allele count SNPs near the high allele count indels; instead, the allele count of the neighboring SNPs closely matches that of the indel. (D) Density of variants (reference bases affected per Mb) in selected genomic regions. (E) Number of indels in coding regions by indel size. Insertions are in red and deletions in blue. Bars representing indel sizes that are a multiple of three are coloured dark red and blue, respectively.

Indels and SNPs are not uniformly distributed across the genome, with autosomal centromeric regions as well as the *X* chromosome containing fewer variants compared to other genomic regions (Figure 1C). Several models have been proposed to explain this pattern, ranging from purifying selection to recurrent hitchhiking to demography [7], [28], [29], although we cannot rule out that low coverage or read mapping quality issues in those regions has affected variant discovery. Moreover, we found that genome-wide SNP, indel, and complex variant densities are strongly correlated on a 1 Mb scale (Spearman's ρ_SNP-del_ = 0.927, ρ_SNP-ins_ = 0.90, ρ_SNP-complex_ = 0.946; Figure S5), suggesting that similar higher-order constraints such as local selection intensities, recombination, or mutation rates may act on all types of variants. To test the impact of recombination, we tested SNP, indel, and complex variant densities against recombination rates (1 cM/Mb), and found that recombination is strongly positively correlated with variant densities (Spearman's ρ_SNPs_ = 0.54, ρ_insertions_ = 0.55, ρ_deletions_ = 0.62, ρ_complex_ = 0.46), which is line with long-held observations regarding the actions of both pervasive positive selection and background selection in *Drosophila* [30], [31]. We further examined the SNP distribution around detected indels in more detail, as it has been proposed that indels may act as “mutators” of surrounding sequences [32], [33]. Consistent with this hypothesis, we observed that the SNP density is elevated in close proximity to indels independent of indel type, dropping quickly to background levels (Figure 2A, 2B, and 2C). On average 26% (40%) of all SNPs per line are within 40 bp (100 bp) of a non-SNP variant. An increase in SNP density around indels has previously been observed between species [32], [33]; here, we present genome-wide evidence of this effect within a single species. Thus, the accuracy of a traditional SNP-calling method based on simple read alignment may suffer materially since a considerable portion of the reads that would contribute to SNP calling may not be mapped correctly as they also contain indels; this problem is circumvented by the integrative variant calling approach employed here.

Interestingly, we observed a similar SNP density increase around other SNPs (Figure 2A), suggesting that either SNPs are also mutagenic or, more likely, that alternative explanations need to be considered for the increased SNP density around variants. We plotted the allele frequencies of SNPs around variants and found that high allele frequency SNPs cluster around other high allele frequency variants (including other SNPs) and, correspondingly, low frequency variants cluster together (Figure 2B and 2C). These findings provide compelling evidence that variants in general are likely not mutagenic, since otherwise we would expect to observe a greater number of rare, thus mostly recent variants, around high frequency alleles. Alternatively, this phenomenon could occur if some genomic regions are more susceptible to mutation than others [32], [34]. We therefore examined the SNP density in the same DNA regions, but in those of the 39 lines which do not contain the focal variants. We found no material enrichment in SNP density in these regions (Figure 2A, 2B, and 2C), providing little evidence of locally increased mutation rate.

### Functional impact of indels and complex variants

Non-coding regions that are conserved between species contain significantly fewer variants than other non-coding regions (Figure 2D, Mann-Whitney U test *P*<2.2e-16), confirming that these regions experience intra-species purifying selection as well. We obtained similar results when integrating histone modification data from the modENCODE Project [9]. We observe fewer indels affecting regulatory genomic regions - especially promoters (marked by tri-methylation of histone H3 at lysine 4, H3K4me3) - than unmarked non-coding regions, (Mann-Whitney U test *P* = 3.1e-14) (Figure S6).

While the majority (>90%) of indels and complex variants fall into intronic or intergenic regions, 80% of all protein-coding genes are affected by indels or complex variants (Table 2). However, only 13% of non-SNP variants within coding regions result in exon disruptions or full gene deletions. More than 50% of indels leading to frame shifts are singletons versus 35% for non-frame shifting indels, indicating that purifying selection is acting against these likely deleterious mutational events. This is illustrated by our observation that the length of indels affecting coding regions is highly biased for sizes that are multiples of three, a pattern that is still visible even up to 51 bp (Figure 2E). Consistent with previous inter-specific comparisons [35], we found that genes affected by disruptive indels or complex variants, are significantly enriched for the functional categories of sensory perception of taste and smell, proteolysis, and innate immune response as well as pathways related to food and drug metabolism (Tables S7 and S8).

**Table 2:** Potential functional indels and complex variants in genes.

Class	Deletions	Insertions	Complex variants	Total
In-frame	5,113 (2,582)	3,001 (1,568)	6,611 (3,614)	14,725 (5,499)
Frameshift	1,514 (1,011)	489 (364)	243 (188)	2,246 (1,313)
Gene disruption	85 (82)	0 (0)	0 (0)	85 (82)
Stop gain	13 (13)	72 (56)	0 (0)	85 (69)
Stop loss	6 (6)	4 (4)	0 (0)	10 (9)
Splice-site disruption	80 (74)	79 (75)	24 (23)	183 (168)
UTR	20,357 (6,728)	15,020 (5,538)	8,026 (4,052)	43,403 (8,783)
Total	27,168 (8,456)	18,665 (6,655)	14,904 (6,533)	60,737 (11,186)

Number in brackets indicates the number of genes affected.

**Figure 3:**
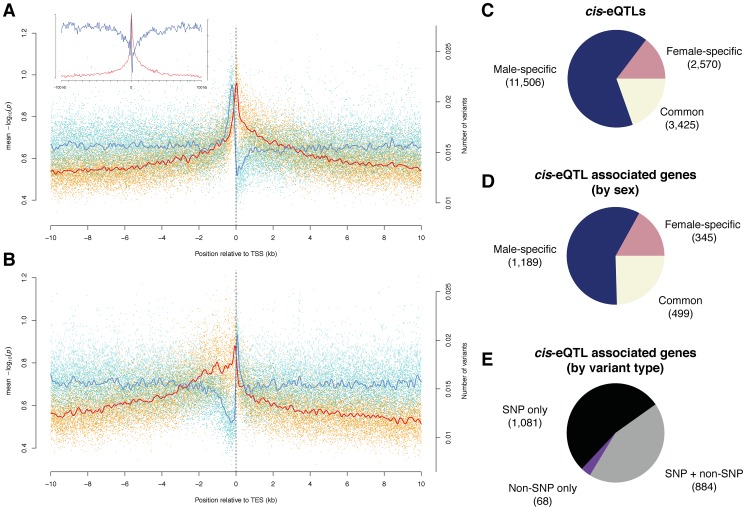
*Cis-*associations of variants with gene expression. (A) and (B) Variant density (blue) and significance of allele associations (red), in males around (A) the transcription start site (TSS) and (B) the transcription end site (TES) averaged out over all transcripts in a 10 kb window. The solid lines are cubic smoothing splines, fit to the data. Transcripts on both strands are orientated such that transcription takes place in the positive direction of the x-axis. The inlet in (A) corresponds to a 100 kb window length. (C) *cis-*eQTLs discovered in males, females, or both sexes (FDR<10%). (D) Breakdown of *cis-*eQTL-associated genes by sex. (E) Breakdown of *cis*-eQTL associated genes, discovered in males or females, by type of variant (*i.e.*, SNP and non-SNP).

Although the density of variants around genes is lower than the overall background, we observed an increase in variant density almost to background levels directly upstream of the transcription start site (TSS), followed by a dramatic drop across the TSS (Figure 3A). This is consistent with strong selective constraint at the TSS but not the region immediately upstream, possibly because this region has high AT content and it is typically depleted of nucleosomes [36], [37]. Indeed, we observed a strong correlation between the local AT content and indel density around the TSS (Figure S7A), strengthening earlier observations that sequence context or chromatin structure may affect the indel rate [38], [39]. We observed a similar, albeit less striking effect at the transcription end site (TES) (Figure 3B and Figure S7B).

### Identification and characterization of *cis*-eQTLs

We used published whole adult microarray gene expression data [6] to perform association analysis between the expression and variants within 10 kb of each gene. We initially identified 9,789 (9,434) genetically variable transcripts (FDR<0.001) in males (females) after removing probes from the microarray analysis that contain genomic variation. We then used the Kruskal-Wallis test followed by permutation-based multiple test correction to find significantly associated variants, termed *cis-*expression QTLs (*cis-*eQTLs), which may point to underlying functional regulatory elements. QTL studies have so far mostly associated bi-allelic variants with gene expression levels [40], [41]; given the availability of a high-resolution, comprehensive catalog of sequence variants, many of which (9.3%) are multi-allelic within the DGRP population, we grouped expression levels according to the corresponding allele as an input to each test. We conducted ∼3.8 million tests for each sex, and restricted our association analysis to variants present in at least three lines. We found 17,501 *cis-*eQTLs in 2,033 genes (26% of the 7,889 genes tested) at a false discovery rate of ∼10% (Figure 3C, 3D, and 3E; Tables S9 and S10; and results for 1% and 20% FDR thresholds are listed in Table S11), generating to our knowledge the first *cis-*eQTL map in *Drosophila*.

**Figure 4:**
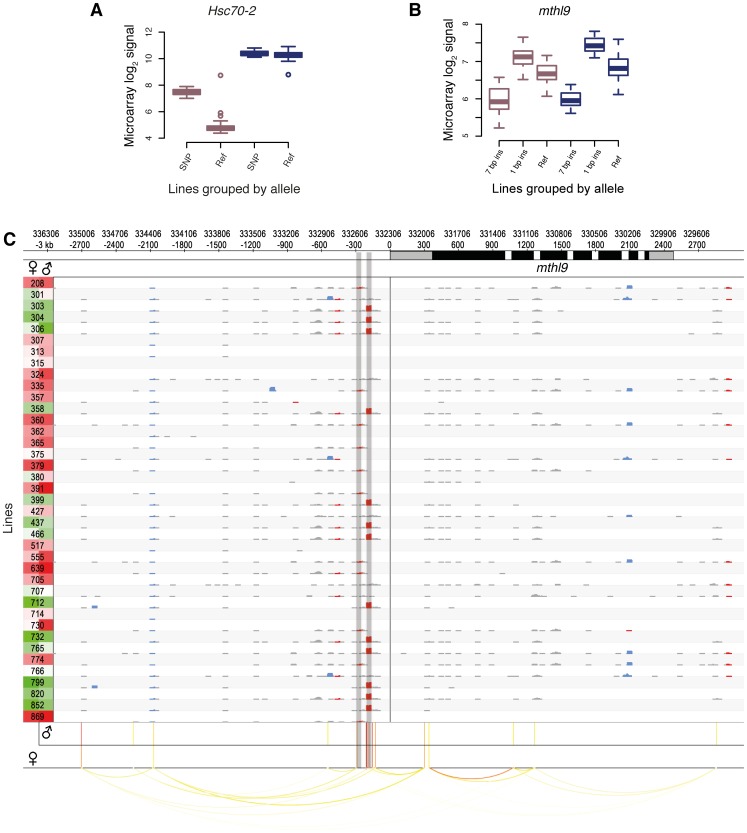
Examples of *cis-*eQTLs and their associated genes. DGRP lines in (A) and (B) are grouped by their allele. Male and female expression levels are depicted in blue and dark pink, respectively. (A) Sex-biased *cis*-eQTL. A SNP (3R:8,875,391) is associated with higher gene expression levels in females only. (B) Indel-based *cis*-eQTLs associated with gene expression. Two insertions (7 bp, 3L:332,512; 1 bp, 3L:332,594, r^2^ = 0.20) are associated with markedly different expression levels in males and females. (C) *cis*-association overview. Plot illustrating the variant and association data for a single gene (*mthl9*) on a rolling window basis. The gene is shown on the top track, with UTRs in grey and coding regions in black. Significant *cis*-eQTLs are drawn below and color-coded by significance for each sex separately (red most significant). Linkage (r^2^>0.5) is shown by arcs, color-coded according to r^2^, with higher values in red. Rows represent all 39 DGRP lines and the left column shows gene expression levels for each line and sex separately (red indicates the highest expression level and green the lowest). The grid contains a representation of variants in rolling 50 bp windows (successive windows overlapping by 45 bp) with net insertions in red, net deletions in blue, and variants not affecting the sequence size (mostly SNPs) in black. The height of each variant indicates the net size of variants with the window, up to 20 bp. The two shaded vertical bars mark the *cis*-eQTLs shown in (B).

Surprisingly, the majority of *cis-*eQTLs were found to be sex-specific (58% specific to males and 17% to females; Figure 3C and 3D, and an example can be found in Figure 4A) with males having more than two-fold more *cis*-eQTL-associated genes compared to females. This result is not an artifact of the significance threshold, as a plot of the underlying association *P*-values in both sexes clearly reveals that the majority of *cis-*eQTLs are sex-specific (Figure S8). Further, the variance in gene expression among females is comparable to that among males as 56% of genetically variable transcripts without any *cis-*eQTL-associations display higher expression variance in females, making it unlikely that such bias affects our findings. In addition, only 38% of transcripts with male-specific *cis*-eQTLs exhibit greater gene expression variance in females than in males, refuting the hypothesis that the more than two-fold greater number of male- compared to female-specific *cis*-eQTL-associated genes may be due to a greater variability in gene expression in females compared to males. Intriguingly, we found that male-specific *cis*-eQTL-associated genes are significantly depleted from the *X* chromosome (*P* = 3.6e-11, χ^2^ test), whereas female-specific *cis*-eQTL (*P* = 0.17) and unbiased (*P* = 0.02) genes show a more uniform chromosomal distribution, perhaps consistent with the observed depletion of male-biased genes on the *X* chromosome [42], [43]. Sex-unbiased *cis*-eQTL-associated genes were found to have more *cis*-eQTLs than sex-biased ones (Figure S9) and the effect of *cis-*eQTLs found in both sexes is larger (Mann-Whitney U test, *P*<2.2e-16; Figure S10). Sex-unbiased *cis-*eQTL-associated genes have also a greater tendency to be expressed only in somatic tissues (Figures S11 and S12), possibly explaining why the underlying variants affect gene expression in both sexes. Nevertheless, more than 20% of sex-specific *cis-*eQTL-associated genes are also only expressed in somatic tissues, indicating that sex-biased changes in gene expression are pervasive [44]. Furthermore, we found that 20% of male-specific *cis-*eQTL-associated genes are exclusively expressed in the testes or the accessory gland in males, whereas the expression of only few (5%) female-specific counterparts are restricted to the ovaries or spermathecae in females. Approximately 60% of male-specific *cis-*eQTL-associated genes are expressed in female reproductive tissues (whereby 28% having greater expression than in any somatic tissue) (Figure S13), yet these genes do not yield any *cis*-eQTLs in females. In other words, despite the fact that these genes are expressed both in males and females, genetic mutations only produce *cis-*eQTLs in males, indicating that these mutations do not significantly affect gene expression in females. Together with the lower number of female-specific *cis*-eQTL-associated genes in general, this suggests that the underlying regulatory architecture may be more constrained in females as compared to males.

We found that 5% of all *cis-*eQTL-associated genes do not exhibit detectable expression in any tissue of the corresponding sex (as represented in FlyAtlas [45]), indicating that the underlying genetic variation induces previously unreported gene expression in young adult flies. These *cis*-eQTLs show an enhanced effect compared to genes previously known to be expressed in adult fly tissues (Figure S14), thus strengthening the finding that these genes become strongly expressed given a specific genetic background. An example of this phenomenon involves *Hsc70-2*. This gene, ranking second among all female-specific associations, is known to be highly expressed in testes of adult males only [45], [46]. However, we find that females from several DGRP lines also exhibit high *Hsc70-2* expression (Figure 4A), an observation which was validated using RNA-seq-based allele imbalance analyses (see below).

Of the *cis-*eQTL-associated genes, 53% are associated with SNPs only, 44% with SNPs and non-SNP variants, and 3% with non-SNP variants only. The latter is expected considering the increased density of SNPs near indels and complex variants (Figure 2A). In total, indels comprise 10% of all *cis*-eQTLs and complex variants 4%, and they have larger effect sizes than SNP *cis-*eQTLs (Mann-Whitney U test, *P* = 1.6e-10; Figure S15). One gene associated with indel *cis*-eQTLs is *mthl9*, where we identified two insertions with markedly different effects on gene expression in both males and females (Figure 4B).

### Genomic distribution of *cis-*eQTLs

Although we considered only *cis-*associations, we identified 1,162 *cis*-eQTLs (6.6%) associated with two genes, and 58 with three genes. We found on average seven (median three) significant *cis*-associations per transcript (males and females combined), with the physical distance between two consecutive *cis*-eQTLs being 689 bp (median 121 bp), thus narrowing putatively causal variants down to a few hundred base pairs. However, *cis-*eQTLs associated with the expression of the same gene were found to be often in strong linkage disequilibrium (average *r^2^* = 0.88).

We examined the genomic location of significant associations relative to TSSs and TESs (Figure 3A and 3B). Both the TSS and TES show the most significant associations independent of the variant type. We observed a quasi-symmetric distribution around TSSs in marked contrast to TESs, where upstream associations are, on average, more significant than downstream associations. These findings are consistent with previous studies in other metazoans including humans [40], [47], [48]. The high density of genetic markers in this study affords however greater resolution, clearly positioning the most significant associations within a window of less than 500 bp around the TSS and 1 kb upstream of the TES, consistent with findings in yeast [49]. The results are also consistent with enrichment for significant associations in H3K4me3 regions (2.8-fold for females and 2.6-fold for males) as compared to the rest of the genome (Table S12). Non-coding conserved regions are not enriched for *cis-*eQTLs, in agreement with findings in humans, where eQTLs are underrepresented in such regions [50]. One explanation could be that variants in conserved non-coding regions that dramatically affected gene expression were purged from the genome. Regions marked by tri-methylated histone H3 at lysine 27 (H3K27me3) are underrepresented for *cis*-eQTLs, consistent with the modification being a heterochromatin mark [51]. However, while we observe an enrichment of *cis*-eQTLs in functionally annotated regions, the majority of *cis*-eQTLs are outside of previously annotated regulatory regions. High-resolution maps of these *cis*-eQTL-derived putative regulatory elements (Figure 4C) may in this regard constitute a powerful resource for dissecting the regulatory architecture of gene expression in further detail.

### Inheritance of gene expression

**Figure 5:**
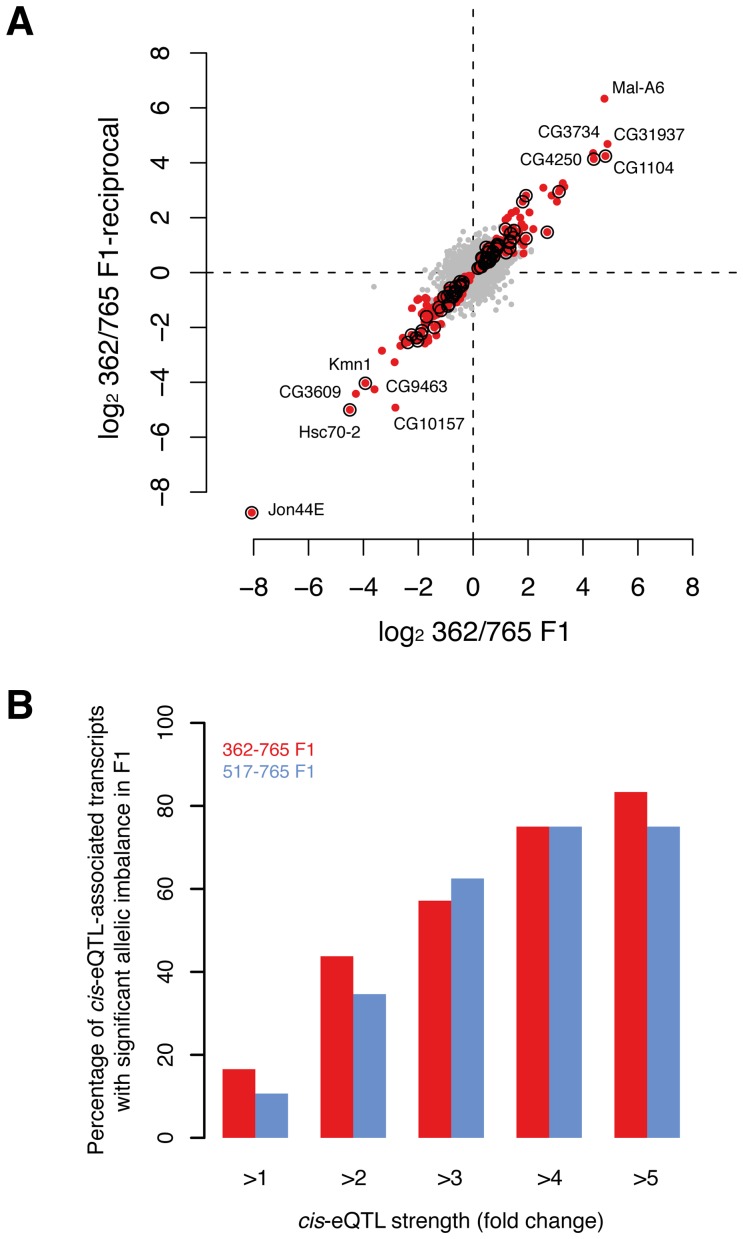
Validation of *cis*-associations in F1. (A) Allelic imbalance measured for ∼7,100 transcripts in F1 (362/765 and reciprocal) with RNA-seq. Dots represent the fold-change (log2) between allele-specific reads counts. Red dots indicate transcripts with significant allelic imbalance in both crosses at a false discovery rate of 10%. Circles mark transcripts that demonstrate significant allelic imbalance and that were found to be associated with *cis*-eQTL in females (note that only *cis*-eQTLs were considered when the allele between both parental lines was not the same). (B) The proportion of *cis*-eQTL-associated transcripts that show allelic expression imbalance in F1s scales with the strength of the *cis*-eQTL (P<0.001, permutation-based, see Methods for details).

To validate *cis*-eQTLs genome-wide, we performed mRNA sequencing (RNA-seq) of reciprocal F_1_ female hybrids from two crosses involving three DGRP lines. Specifically, we aimed to identify whether transcripts predicted to be regulated by *cis*-eQTLs exhibit a significant allele-specific bias in gene expression. Since both alleles act in the cross in the same *trans* environment, differential expression in the F_1_ is a direct measure of *cis*-regulatory activity [52], [53]. As a quality control step, we first analyzed the correlation of the expression data between the reciprocal crosses, obtaining a Spearman correlation coefficient of >99% for both F1_362–765-initial_/F1_362–765-reciprocal_ and F1_517–765-initial_/F1_517–765-reciprocal_, respectively. We then tested on average ∼7,100 transcripts for allelic imbalance in the two crosses. Using a 10% FDR, we found that 6% (443) of the tested transcripts show significant allele-specific gene expression (Figure 5A) with a median allelic expression difference of 1.5-fold. Of the *cis*-eQTLs for which the parental lines have different alleles, and whose population mean effect on microarray expression was two-, three- and four-fold among all 39 lines, we found that on average 39%, 60% and 75% of the associated transcripts respectively exhibited a significant allelic imbalance in the crosses (Figure 5B). These results are in line with similar, albeit smaller-scale analyses in yeast [49] and mouse [48], and thus provide support for our *cis*-eQTL map, revealing that the majority of strong cis-eQTLs can be validated in heterozygous individuals.

## Discussion

We present a comprehensive, genome-wide list of variants of all major types (*i.e.*, single-, multi-nucleotide-, and structural variation) for a *Drosophila* wild-derived population, significantly expanding the catalog of variants that has been compiled to date for this model organism (*e.g.*, [7], [13]–[15], [20]). Our analyses reveal extensive non-SNP variation among lines, with a high indel frequency (roughly every 150 bp) and indels in 80% of the protein-coding genes. The majority of indels do not alter the reading frame, but nevertheless reveal surprising tolerance for indels in coding regions. Our data are relevant to the inference of underlying mechanisms of varying mutation rates across genomes [32]. We show elevated SNP density around other SNPs as well as indels; that neighboring variants have similar allele frequencies; and that regions with indels are not particularly mutation-prone. These data suggest that this phenomenon reflects the existence of small haploblocks. While these may arise because of segregating mutational hotspots, a more likely explanation invokes the demographic history of the population. For example, admixture may generate such a haplotype structure to an extent largely dictated by the local genomic recombination environment [54] – potentially consistent with our observed correlation between recombination rate and variant density. Thus, while our data suggest that these population level processes may lead to incorrect inference regarding the mutagenic nature of indels, this will be a topic of further investigation in future studies during which alternative explanations (*e.g.*, duplication hotspots) will also be explored.

The presented variant collection constitutes a useful resource for several scientific disciplines, including phylogenetics and population genetics, which are beyond the scope of this study. Here, we applied this resource to study the impact of natural polymorphisms on gene expression, identifying *cis*-eQTLs for >2,000 genes (10% FDR). Although further work is necessary to identify the causal variants underlying *cis*-eQTLs, the combined results provide general insights into the complexity and evolution of the *cis-*regulatory architecture of gene expression in *Drosophila*. More specifically, the data suggest that gene expression variation is governed by multiple *cis-*eQTLs of different variant types, that the most significant *cis-*eQTLs are tightly clustered around the TSS and immediately upstream of the TES, but that many *cis-*eQTLs are also located in regions currently devoid of regulatory annotations. These regions likely play an important role in gene regulation as their alteration has an expression impact that can be observed in whole adult fly expression profiles. In addition, the data suggest that the regulatory architecture is partly decoupled between males and females, at least within the tested settings, as underlying changes predominantly affect gene expression in males. However, *cis*-eQTLs common to both sexes typically have larger effects. This supports the hypothesis that intersexual ontogenetic conflict in *D. melanogaster* may stem from many sexually antagonistic alleles with low effect sizes [55]. Finally, the detection of many sex-specific *cis-*eQTLs indicates that genetic factors play an important role in sex-biased gene expression as changes in *cis*-regulatory sequences appear to significantly contribute to this phenomenon. While the relative significance of these changes and *cis*-regulatory variation in general in governing morphological evolution remains an important topic of debate [56], [57], we believe that the presented catalog of *cis*-regulatory changes constitutes a valuable resource to further illuminate this discussion.

## Materials and Methods

### Variant prediction

We used Release 5 of the *Drosophila melanogaster* reference genome for all analyses.

#### Stage 1: Variant discovery

We used PrInSeS-G [19], in conjunction with BWA [58] to perform the initial variant discovery using whole-genome Illumina sequencing reads.

#### Stage 2: Variant refinement

We computed whole-genome alignments between the chromosomes of each line and the reference genome in order to reduce the amount of variant fragmentation and to optimize the representation of variants among lines (see Text S1).

#### Stage 3: Variant genotyping

The purpose of genotyping is to improve variant discovery for DGRP lines or genomic regions whose sequencing coverage was originally too low for effective *de novo* assembly by PrInSeS-G. The algorithm is described in detail in Text S1, and Figure S16.

### Gene expression analysis

Whole-adult gene expression microarray raw data files (*i.e.*, .CEL) for 39 inbred lines were downloaded from the EBI ArrayExpress Archive (accession number E-MEXP-1594). Briefly, Ayroles *et al.* [6] derived inbred lines from the Raleigh (USA) population by 20 generation full-sib mating and hybridized RNA from two independent pools (25 flies/line/sex) on Affymetrix *Drosophila* 2.0 microarrays. The raw data set consists of gene expression measurements for males and female in two replicates. We used a four-step pipeline to analyse the microarray data:

#### Step 1

We obtained a custom probe set definition file (http://brainarray.mbni.med.umich.edu/Brainarray/Database/CustomCDF/13.0.0/refseq.asp) for the Affymetrix *Drosophila* 2.0 platform, which is based on an updated set of 20,666 transcripts (UCSC RefSeq; June 22, 2010). We excluded probes that overlap with any type of sequence variants and probe sets that were covered by less than three probes after probe removal. In addition, we verified whether polymorphic duplicate regions may have contributed to gene expression variation. We aligned all insertions (>100 bp) to the annotated *D. melanogaster* transcriptome with the alignment program blat, retaining only high sequence similarity hits (>90%), since those would be the most likely candidates to cause problems with cross-hybridization on microarrays. We found only two insertions that could be mapped against annotated transcripts with both insertions targeting the same exon, demonstrating that duplication does not constitute an important confounding factor.

#### Step 2

Raw microarray data were normalized across all sexes and lines with the Robust Multichip Average (RMA) algorithm as implemented in the *R affy* package (default settings).

#### Step 3

We removed transcripts that were not or lowly expressed among lines using the Wilcoxon signed rank-based gene expression presence/absence detection algorithm (*affy* package, *R*). A transcript was classified as expressed in a single line if it was detected in either one or both sexes, respectively, and the requirement to be detected in both replicates. Finally, we only kept 16,985 probe sets (or 10,347 genes) that were classified as expressed in at least 4 out of 39 lines.

#### Step 4

We used ANOVA on each transcript and sex separately to test whether it is genetically variable, *i.e.*, has a significant line term, under a conservative FDR of 0.001 [6]. In total, we found 9,789 (6,239) and 9,434 (5,797) genetically variable transcripts (genes) in males and females, respectively, of which 6,745 (4,147) were variable in both sexes.

### Tissue-specific gene expression

Tissue-specific gene expression data for young adult flies was obtained from FlyBase (ftp://flybase.org/flybase/associated_files/Gelbart.2010.10.13.tar.gz). A gene was considered to be expressed in a tissue if the expression level was above 50.

### *Cis*-associations

We grouped overlapping variants for all 39 lines as alternative alleles. For each such group of variants, we calculated associations with any transcript whose either end is within 10 kb, and for which microarray expression data was available. We employed two statistical methods: the first one, *Alignment Score Association*, is linear regression between the rank of alignment scores of all alleles and the rank of expression; in this context we defined alignment score to be the maximum of the number of bases inserted and that removed by the variant. Note that, since we used a non-parametric model, there was little advantage of using a more complex alignment score. This method thus associates the size of the variants rather than the exact genotype, which is important for the cases where more than two alleles have variants of different lengths. For the second method, *Allele Association*, we grouped the rank of expression of each line by its allele at each variant locus; we then performed a Kruskal-Wallis test. For both methods we corrected for multiple tests by repeating each test for 10,000 random permutations of gene expression, in a similar fashion to [40]. We then took for each permutation the lowest *P*-value for all tests in the same transcript, thus obtaining a vector of 10,000 such *P*-values. This vector was then sorted, and we obtained the adjusted *P*-value threshold from the value indexed by our unadjusted threshold. For example, the adjusted (multiple-test corrected) *P*-value threshold for a nominal threshold of 0.05 is the 500'th element of the sorted vector (10,000×0.05 = 500). The FDR is the ratio of the number of transcripts expected to pass the adjusted *P-*value threshold by chance over the number that actually passes. This, of course, implies that a different adjusted threshold is used for each transcript for the same nominal threshold. Further, since the permutations are random, each run of the above workflow will produce a slightly different list of eQTLs, since the *P*-value thresholds will be slightly different each time. We note that the overlap between the two association methods was more than 95% for all metrics, as both give approximately the same significance measure for bi-allelic variants, hence we only presented the results of the Allele Association method in the main manuscript.

### Variant validation

We used five complementary methods to validate our variant calls. First, we compared our variant catalogue against publicly available indel data from the FLYSNPdb database, which features indels (1 bp to 360 bp) from five previously established *D. melanogaster* lines (but none of the DGRP lines) [20]. Second, we compared our calls with those published by Mackay *et al.* [7]. We used two methods (comparing all variants and comparing SNPs not within 30 bps of non-SNPs, Table S1). In many cases, SNP calls by the other study overlapped or were close to indel calls, which indicates a possible false positive since methods based on direct read alignment are confounded by indels. Third, we aligned Roche-454 whole genome sequencing reads from Mackay *et al.* available for all 39 lines [7] to validate our SNP calls (Massouras *et al.*, in preparation), including those that (a) were originally supported by two thirds or more of the Illumina reads and (b) were covered by at least two Roche-454 reads (Table S2). Fourth, we sequenced mRNA from three DGRP lines at the young adult stage (see below and main manuscript for results) (Table S3). Finally, we examined 92 large indels (200 bp to 5.6 kb) in five DGRP lines using PCR after which we randomly selected 10 for subsequent Sanger sequencing to validate the predicted variant breakpoints (Table S4).

### Variant validation using RNA–seq and allelic expression imbalance in F1

All fly stocks were grown at 25°C and a 12 h light-dark cycle on corn-meal fly medium. We collected virgin flies from three of the DGRP lines (lines 362, 517, and 765) during three days and we set up the following crosses: ♀ 362×♂ 517 (and reciprocal) and ♀ 517×♂ 765 (and reciprocal). We performed RNA-seq to assess transcript expression profiles of 3–5 day old F1 females. 25 flies per sample were frozen between 1–3 pm. Total RNA was extracted using the combined Trizol/RNeasy (Qiagen, http://www.qiagen.com/) protocol. RNA quality was measured using RNA Labchips and Bioanalyzer from Agilent Technologies (http://www.chem.agilent.com/). RNA-seq libraries were prepared using the RNA-True seq kit (Illumina). Prepared libraries were sequenced with an Illumina Genome Analyzer 2 DNA Sequencing Platform (GTF, Lausanne). We used the UCSC transcript annotation to derive the sequences of the annotated transcriptome from the reference genome. We mapped the reads to both the genomes and the annotated transcriptomes; any reads aligning to more than one genomic mapping coordinate were discarded. The tool we built specifically for this purpose takes as input the sequencing reads, two genome haplotypes, and the UCSC-annotated transcript coordinates in order to derive two transcriptomes. For every read in turn it attempts to align it to both transcriptome haplotypes. Reads that align to the same position in a transcript of the two haplotypes are then checked to see if the alignment overlaps with any variants. If it does, and the variants are different between the two haplotypes, the read is deemed to “support” the allele that produces the lowest number of mismatches. If the read aligns to only one transcriptome haplotype, it is also deemed to support the corresponding allele. In this way the tool measures allele-specific differential gene expression in the same line.

For the parental lines, we used the aforementioned tool, supplying it with the Stage 3 genome (see Variant prediction paragraph for more details) and the reference genome. For the crosses we gave it the two parental Stage 3 genomes. In each case, for each position with a variant we counted the number of reads best aligning to each allele. As a result, for the parental lines, this method provides a measurement of the true positive and false positive rate with regard to our variant discovery, since it measures the differential alignment of reads to the reference sequence and the target haplotype of the line. For the F1s, this method provides a measurement for differential expression for each transcript.

For variant validation, we considered a variant true positive (TP) when either all aligned reads support this variant, as opposed to the reference or when the number of reads that support it compared to that supporting the reference results in *P*<0.05 in a two-tailed binomial test. We considered a variant a false positive (FP) when either all aligned reads supported the reference at this position, or the number supporting the reference was significant using the same binomial test.

### Allelic expression imbalance analysis

We tested for significant allelic expression imbalance between alleles in F1 using binomial exact tests (two-sided). About half of all tested transcripts were covered by at least 200 discriminative reads, thus providing a reasonable power to detect even small allelic imbalances (≥1.5-fold allelic expression differences). *P*-values were corrected for multiple hypothesis testing with the Benjamini & Hochberg procedure (implemented in the function *p.adjust*, R). Only transcripts that were tested for *cis*-associations in females and that were covered by at least 20 informative reads (*i.e.*, allele1+allele2≥20) were tested for significant allelic imbalance. Transcripts that passed with 10% FDR in both reciprocal crosses were considered to have a significant allelic imbalance in gene expression.

The increase in percent-validated (Figure 5B) was tested by permutation analysis. For each “*cis*-eQTL strength cutoff” we obtained a random set of transcripts (from all tested *cis*-eQTL transcripts) and calculated how many of those where validated in F1. We repeated this sampling procedure 1,000 times and obtained an empirical P-value for each “*cis*-eQTL strength cutoff” by counting how many random transcript sets scored higher than the real set of transcripts and divided by the number of permutations (*i.e.*, 1,000).

### Recombination estimates

The Fiston-Lavier [59] recombination rate calculator was used to estimate the recombination rate (cM/Mb) in 1 Mb windows along each chromosome (only chromosome 4 excluded). The rate at the center of each interval was used.

### Genomic distribution of *cis-*eQTL–associated genes

Genes were grouped into four different categories (*i.e.*, genes with no-, male-specific-, female-specific-, and sex-unbiased *cis*-eQTLs) and within each category the percentage of genes located on the *X* chromosome was calculated. For *cis*-eQTL associated genes, we calculated whether the fraction of *X*-linked genes deviates significantly from non-*cis*-eQTL associated genes using the Chi-square test with Yates' correction.

### Data availability

The full catalog of variants can be obtained from dgrp.epfl.ch/downloads. The RNA-seq data are available in the ArrayExpress Database using accession number E-MTAB-1266.

## Supporting Information

Figure S1 Results of variant genotyping. Size (reference bases in thousands, left y-axis) of variants after Stage 2 (re-alignment, yellow) and Stage 3 (genotyping, blue), ordered by sequencing coverage (red line, right y-axis). This figure illustrates that the genotyping stage improves the consistency of variant calling materially, particularly for low-coverage genomes.
(PDF)

Figure S2 Number of variants by minor allele count. The number of insertions, deletions, complex variants and segmental duplications/copy number variants discovered is plotted next to the number expected under the neutral hypothesis. Deletions and especially segmental duplications are more enriched for low allele counts, which suggests that they are more under negative selection than the other variant types.
(PDF)

Figure S3 Allele-frequency spectrum of non-SNP variants by genomic location.
(PDF)

Figure S4 Evolutionary conservation analysis of insertions in the *Rh6* coding sequence. Both insertions were predicted in all 39 lines and are also present in seven out of eleven *Drosophila* species other than *D. melanogaster*. Moreover, the resulting gene model supports an *Rh6* cDNA clone of the OregonR/white strain. Thus, the reference genome has a likely rare allele, which disrupts a splice site and introduces a premature stop codon resulting in a truncated protein.
(PDF)

Figure S5 Genome-wide correlation between SNP and non-SNP densities. The concentration (variants per kb) of SNPs is correlated with deletions (blue), insertions (red), and complex variants (orange). Densities were calculated in non-overlapping genomic bins of 50 kb across all autosomes and the *X* chromosome.
(PDF)

Figure S6 Variant concentration in histone modified regions. The histone marks for adult flies were obtained from modENCODE. SNPs are in black/grey, indels and complex variants in purple.
(PDF)

Figure S7 Variant density and AT content (A) near TSS, (B) near TES. The inlet shows the same plot between 10 kb up- and downstream of respectively the TSS and TES (spline-smoothed). Blue dots depict variant density and red dots AT content (%).
(PDF)

Figure S8 Comparison of *P*-values for sex-specific associations. (a) Female-specific and (b) male-specific association *P*-values have mostly no significant counterpart for the other sex, ruling out the possibility that they labeled sex-specific only for marginally failing to meet the significance threshold.
(PDF)

Figure S9 Correlation between sex-bias and number of *cis*-associations. Transcripts were grouped by the number of *cis*-eQTLs and the y-axis indicates the percentage of transcripts for which *cis*-associations were detected in one sex only. Linear regression fits are plotted for both sexes separately.
(PDF)

Figure S10 Effect size of sex-biased and unbiased *cis*-eQTLs.
(PDF)

Figure S11 Tissue-specific gene expression pattern analysis of *cis*-eQTL-associated genes. Scatter plots showing the highest expression (log2) of *cis*-eQTL-associated genes in 12 somatic tissues (X-axis) and either male-specific (*i.e.*, testis or accessory gland; Y-axis, upper panels) or female-specific tissues (*i.e.*, ovary or spermatheca; Y-axis, lower panels). Dashed horizontal and vertical lines denote the expression level at which we considered transcripts as expressed (see Methods for details).
(PDF)

Figure S12 Venn diagrams depicting the percentage of *cis*-eQTL-associated genes that are expressed either in somatic tissues only, sex-specific tissues only, or both. Genes not expressed in any tissue or for which tissue-specific gene expression data was missing were not considered in this analysis.
(PDF)

Figure S13 Bar graphs showing the number of *cis*-eQTL-associated genes that exhibit highest expression in the respective tissues in either males (*i.e.*, including testes and accessory gland; upper panels) or females (*i.e.*, including ovary and spermatheca; lower panels). Genes classified as being not expressed in any tissue are denoted as ‘not-detected.’
(PDF)

Figure S14 *cis*-eQTL effect size by category (*i.e.*, sex-unbiased, male-, or female-specific) and by their gene expression status among different tissues [45]. M+: expressed in males; M−: not expressed in males; F+: expressed in females; F−: not expressed in females.
(PDF)

Figure S15 Effect size of *cis*-eQTLs by variant type (*i.e.*, SNP, indel, complex variant) and indel size.
(PDF)

Figure S16 Illustration of differential read pair alignment for variant imputation. In this example, there are three alleles discovered after the first two stages of variant calling in the population. Read pairs are aligned to all alleles and the reference sequence. Reads r1' and r2 best align to allele 2, *i.e.*, they either only align to this allele or produce the lowest number of mismatches when aligned to allele 2 compared to the alternatives. Thus allele 2 receives two positive and zero negative votes, while the other two alleles receive zero positive and two negative votes. A new variant call is made when the positive exceed the negative votes by at least one. An existing variant call (*i.e.*, a variant called in stages 1 and 2) is removed when the positive votes are not greater than half the negative votes. In all cases the votes are recorded as tags in the variant list.
(PDF)

Table S1 Comparison of SNP calls to those by Mackay et al., Nature, 2012.
(XLSX)

Table S2 Validation of SNPs using Roche-454 whole-genome sequencing data.
(XLSX)

Table S3 Validation of variants in exons by RNA-Seq.
(XLSX)

Table S4 Validation of 92 variants >200 bp using PCR in five DGRP lines.
(XLSX)

Table S5 Allele count frequency distribution by variant type. Average number of variants per line.
(XLSX)

Table S6 Evolutionary conservation analysis of 30 indels. This table contains information about the ancestral allele state of all indels that were predicted in 39 lines and which intersected with protein coding sequences. Multiple sequence alignment around the indel position was obtained from the UCSC genome browser (database: dm3, table: multiz15way) and D. simulans, D. yakuba, and D. ananassae were used as an outgroup.
(XLSX)

Table S7 Functional enrichment (GO) for genes affected by disruptive non-SNP variants. Gene ontology enrichment (biological processes) was tested with DAVID (http://david.abcc.ncifcrf.gov/). Multiple hypothesis testing was performed with the Benjamini & Hochberg correction procedure and a FDR of 0.05.
(XLSX)

Table S8 KEGG pathways for genes affected by disruptive non-SNP variants. Enrichment in KEGG pathways was tested with DAVID (http://david.abcc.ncifcrf.gov/). Multiple hypothesis testing was performed with the Benjamini & Hochberg correction procedure and a FDR of 0.05.
(XLSX)

Table S9 List of significant associations detected in females.
(XLSX)

Table S10 List of significant associations detected in males.
(XLSX)

Table S11 Comparison of cis-EQTLs by false discovery rate threshold.
(XLSX)

Table S12 Concentration of variants and cis-eQTLs in selected regions. Histone marks for adult females and males were obtained from modENCODE.
(XLSX)

Text S1 Supplementary Information.
(DOC)

## References

[1] Mackay TFC, Stone EA, Ayroles JF (2009) The genetics of quantitative traits: challenges and prospects. Nat Rev Genet 10: 565–577. 19584810 10.1038/nrg2612

[2] Consortium H (2010) Integrating common and rare genetic variation in diverse human populations. Nature 467: 52–58. 20811451 10.1038/nature09298 PMC3173859

[3] Mills RE, Walter K, Stewart C, Handsaker RE, Chen K, et al (2011) Mapping copy number variation by population-scale genome sequencing. Nature 470: 59–65. 21293372 10.1038/nature09708 PMC3077050

[4] Keane TM, Goodstadt L, Danecek P, White MA, Wong K, et al (2011) Mouse genomic variation and its effect on phenotypes and gene regulation. Nature 477: 289–294. 21921910 10.1038/nature10413 PMC3276836

[5] Gan X, Stegle O, Behr J, Steffen JG, Drewe P, et al (2011) Multiple reference genomes and transcriptomes for Arabidopsis thaliana. Nature 477: 419–423. 21874022 10.1038/nature10414 PMC4856438

[6] Ayroles JF, Carbone MA, Stone EA, Jordan KW, Lyman RF, et al (2009) Systems genetics of complex traits in Drosophila melanogaster. Nat Genet 41: 299–307. 19234471 10.1038/ng.332 PMC2752214

[7] Mackay TFC, Richards S, Stone EA, Barbadilla A, Ayroles JF, et al (2012) The Drosophila melanogaster Genetic Reference Panel. Nature 482: 173–178. 22318601 10.1038/nature10811 PMC3683990

[8] Rubin GM, Lewis EB (2000) A Brief History of Drosophila's Contributions to Genome Research. Science 287: 2216–2218. 10731135 10.1126/science.287.5461.2216

[9] Consortium Tm, Roy S, Ernst J, Kharchenko PV, Kheradpour P, et al (2010) Identification of Functional Elements and Regulatory Circuits by Drosophila modENCODE. Science 330: 1787–1797. 21177974 10.1126/science.1198374 PMC3192495

[10] Hens K, Feuz J-D, Isakova A, Iagovitina A, Massouras A, et al (2011) Automated protein-DNA interaction screening of Drosophila regulatory elements. Nat Meth 8: 1065–1070. 10.1038/nmeth.1763 PMC3929264 22037703

[11] Adams MD, Celniker SE, Holt RA, Evans CA, Gocayne JD, et al (2000) The Genome Sequence of Drosophila melanogaster. Science 287: 2185–2195. 10731132 10.1126/science.287.5461.2185

[12] Moriyama EN, Powell JR (1996) Intraspecific nuclear DNA variation in Drosophila. Mol Biol Evol 13: 261–277. 8583899 10.1093/oxfordjournals.molbev.a025563

[13] Emerson JJ, Cardoso-Moreira M, Borevitz JO, Long M (2008) Natural Selection Shapes Genome-Wide Patterns of Copy-Number Polymorphism in Drosophila melanogaster. Science 320: 1629–1631. 18535209 10.1126/science.1158078

[14] Cridland JM, Thornton KR (2010) Validation of rearrangement break points identified by paired-end sequencing in natural populations of Drosophila melanogaster. Genome Biol Evol 2: 83–101. 20333226 10.1093/gbe/evq001 PMC2839345

[15] Langley CH, Stevens K, Cardeno C, Lee YCG, Schrider DR, et al (2012) Genomic Variation in Natural Populations of Drosophila melanogaster. Genetics 10.1534/genetics.112.142018 PMC3454882 22673804

[16] Rebeiz M, Pool JE, Kassner VA, Aquadro CF, Carroll SB (2009) Stepwise Modification of a Modular Enhancer Underlies Adaptation in a Drosophila Population. Science 326: 1663–1667. 20019281 10.1126/science.1178357 PMC3363996

[17] Wittkopp PJ, Kalay G (2012) Cis-regulatory elements: molecular mechanisms and evolutionary processes underlying divergence. Nat Rev Genet 13: 59–69. 10.1038/nrg3095 22143240

[18] Carbone MA, Jordan KW, Lyman RF, Harbison ST, Leips J, et al (2006) Phenotypic variation and natural selection at Catsup, a pleiotropic quantitative trait gene in Drosophila. Current Biology 16: 912–919. 16682353 10.1016/j.cub.2006.03.051 PMC10766118

[19] Massouras A, Hens K, Gubelmann C, Uplekar S, Decouttere F, et al (2010) Primer-initiated sequence synthesis to detect and assemble structural variants. Nat Meth 7: 485–486. 10.1038/nmeth.f.308 20543844

[20] Chen D, Ahlford A, Schnorrer F, Kalchhauser I, Fellner M, et al (2008) High-resolution, high-throughput SNP mapping in Drosophila melanogaster. Nat Meth 5: 323–329. 10.1038/nmeth.1191 18327265

[21] Yalcin B, Wong K, Agam A, Goodson M, Keane TM, et al (2011) Sequence-based characterization of structural variation in the mouse genome. Nature 477: 326–329. 21921916 10.1038/nature10432 PMC3428933

[22] Mills RE, Pittard WS, Mullaney JM, Farooq U, Creasy TH, et al (2011) Natural genetic variation caused by small insertions and deletions in the human genome. Genome Research 21: 830–839. 21460062 10.1101/gr.115907.110 PMC3106316

[23] Cao J, Schneeberger K, Ossowski S, Gunther T, Bender S, et al (2011) Whole-genome sequencing of multiple Arabidopsis thaliana populations. Nat Genet 43: 956–963. 21874002 10.1038/ng.911

[24] A map of human genome variation from population-scale sequencing. Nature 467: 1061–1073. 10.1038/nature09534 PMC3042601 20981092

[25] Kim PM, Lam HYK, Urban AE, Korbel JO, Affourtit J, et al (2008) Analysis of copy number variants and segmental duplications in the human genome: Evidence for a change in the process of formation in recent evolutionary history. Genome Research 18: 1865–1874. 18842824 10.1101/gr.081422.108 PMC2593581

[26] Fiston-Lavier A-S, Anxolabehere D, Quesneville H (2007) A model of segmental duplication formation in Drosophila melanogaster. Genome Research 17: 000. 10.1101/gr.6208307 PMC1987339 17726166

[27] Schrider Daniel R, Hourmozdi Jonathan N, Hahn Matthew W (2011) Pervasive Multinucleotide Mutational Events in Eukaryotes. Current Biology 21: 1051–1054. 21636278 10.1016/j.cub.2011.05.013 PMC4744473

[28] Betancourt AJ, Kim Y, Orr HA (2004) A pseudohitchhiking model of X vs. autosomal diversity. Genetics 168: 2261–2269. 15611190 10.1534/genetics.104.030999 PMC1448734

[29] Andolfatto P (2001) Contrasting patterns of X-linked and autosomal nucleotide variation in Drosophila melanogaster and Drosophila simulans. Mol Biol Evol 18: 279–290. 11230529 10.1093/oxfordjournals.molbev.a003804

[30] Begun DJ, Aquadro CF (1992) Levels of naturally occurring DNA polymorphism correlate with recombination rates in D. melanogaster. Nature 356: 519–520. 1560824 10.1038/356519a0

[31] Charlesworth B, Morgan MT, Charlesworth D (1993) The effect of deleterious mutations on neutral molecular variation. Genetics 134: 1289–1303. 8375663 10.1093/genetics/134.4.1289 PMC1205596

[32] Hodgkinson A, Eyre-Walker A (2011) Variation in the mutation rate across mammalian genomes. Nat Rev Genet 12: 756–766. 21969038 10.1038/nrg3098

[33] Tian D, Wang Q, Zhang P, Araki H, Yang S, et al (2008) Single-nucleotide mutation rate increases close to insertions/deletions in eukaryotes. Nature 455: 105–108. 18641631 10.1038/nature07175

[34] McDonald MJ, Wang W-C, Huang H-D, Leu J-Y (2011) Clusters of Nucleotide Substitutions and Insertion/Deletion Mutations Are Associated with Repeat Sequences. PLoS Biol 9: e1000622 doi:10.1371/journal.pbio.1000622.. 21697975 10.1371/journal.pbio.1000622 PMC3114760

[35] Clark AG, Eisen MB, Smith DR, Bergman CM, Oliver B, et al (2007) Evolution of genes and genomes on the Drosophila phylogeny. Nature 450: 203–218. 17994087 10.1038/nature06341

[36] Mavrich TN, Jiang C, Ioshikhes IP, Li X, Venters BJ, et al (2008) Nucleosome organization in the Drosophila genome. Nature 453: 358–362. 18408708 10.1038/nature06929 PMC2735122

[37] Peckham HE, Thurman RE, Fu Y, Stamatoyannopoulos JA, Noble WS, et al (2007) Nucleosome positioning signals in genomic DNA. Genome Research 17: 1170–1177. 17620451 10.1101/gr.6101007 PMC1933512

[38] Tolstorukov MY, Volfovsky N, Stephens RM, Park PJ (2011) Impact of chromatin structure on sequence variability in the human genome. Nat Struct Mol Biol 18: 510–515. 21399641 10.1038/nsmb.2012 PMC3188321

[39] Tanay A, Siggia ED (2008) Sequence context affects the rate of short insertions and deletions in flies and primates. Genome Biol 9: R37. 18291026 10.1186/gb-2008-9-2-r37 PMC2374710

[40] Stranger BE, Forrest MS, Dunning M, Ingle CE, Beazley C, et al (2007) Relative Impact of Nucleotide and Copy Number Variation on Gene Expression Phenotypes. Science 315: 848–853. 17289997 10.1126/science.1136678 PMC2665772

[41] Cheung VG, Nayak RR, Wang IX, Elwyn S, Cousins SM, et al (2010) Polymorphic *Cis-* and *Trans*-Regulation of Human Gene Expression. PLoS Biol 8: e1000480 doi:10.1371/journal.pbio.1000480.. 20856902 10.1371/journal.pbio.1000480 PMC2939022

[42] Parisi M, Nuttall R, Naiman D, Bouffard G, Malley J, et al (2003) Paucity of Genes on the Drosophila X Chromosome Showing Male-Biased Expression. Science 299: 697–700. 12511656 10.1126/science.1079190 PMC1363366

[43] Bachtrog D, Toda NRT, Lockton S (2010) Dosage Compensation and Demasculinization of X Chromosomes in Drosophila. Current Biology 20: 1476–1481. 20705467 10.1016/j.cub.2010.06.076 PMC4511158

[44] Zhang Y, Sturgill D, Parisi M, Kumar S, Oliver B (2007) Constraint and turnover in sex-biased gene expression in the genus Drosophila. Nature 450: 233–237. 17994089 10.1038/nature06323 PMC2386141

[45] Chintapalli VR, Wang J, Dow JAT (2007) Using FlyAtlas to identify better Drosophila melanogaster models of human disease. Nature Genetics 39: 715–720. 17534367 10.1038/ng2049

[46] Graveley BR, Brooks AN, Carlson JW, Duff MO, Landolin JM, et al (2011) The developmental transcriptome of Drosophila melanogaster. Nature 471: 473–479. 21179090 10.1038/nature09715 PMC3075879

[47] Veyrieras J-B, Kudaravalli S, Kim SY, Dermitzakis ET, Gilad Y, et al (2008) High-Resolution Mapping of Expression-QTLs Yields Insight into Human Gene Regulation. PLoS Genet 4: e1000214 doi:10.1371/journal.pgen.1000214.. 18846210 10.1371/journal.pgen.1000214 PMC2556086

[48] Doss S, Schadt EE, Drake TA, Lusis AJ (2005) Cis-acting expression quantitative trait loci in mice. Genome Res 15: 681–691. 15837804 10.1101/gr.3216905 PMC1088296

[49] Ronald J, Brem RB, Whittle J, Kruglyak L (2005) Local Regulatory Variation in Saccharomyces cerevisiae. PLoS Genet 1: e25 doi:10.1371/journal.pgen.0010025. 16121257 10.1371/journal.pgen.0010025 PMC1189075

[50] Stranger BE, Nica AC, Forrest MS, Dimas A, Bird CP, et al (2007) Population genomics of human gene expression. Nat Genet 39: 1217–1224. 17873874 10.1038/ng2142 PMC2683249

[51] Mikkelsen T, Ku M, Jaffe D, Issac B, Lieberman E, et al (2007) Genome-wide maps of chromatin state in pluripotent and lineage-committed cells. Nature 448: 553–560. 17603471 10.1038/nature06008 PMC2921165

[52] McManus CJ, Coolon JD, Duff MO, Eipper-Mains J, Graveley BR, et al (2010) Regulatory divergence in Drosophila revealed by mRNA-seq. Genome Res 20: 816–825. 20354124 10.1101/gr.102491.109 PMC2877578

[53] Wittkopp P, Haerum B, Clark A (2004) Evolutionary changes in cis and trans gene regulation. Nature 430: 85–88. 15229602 10.1038/nature02698

[54] Pfaff CL, Parra EJ, Bonilla C, Hiester K, McKeigue PM, et al (2001) Population structure in admixed populations: effect of admixture dynamics on the pattern of linkage disequilibrium. Am J Hum Genet 68: 198–207. 11112661 10.1086/316935 PMC1234913

[55] Chippindale AK, Gibson JR, Rice WR (2001) Negative genetic correlation for adult fitness between sexes reveals ontogenetic conflict in Drosophila. Proceedings of the National Academy of Sciences 98: 1671–1675. 10.1073/pnas.041378098 PMC29315 11172009

[56] Hoekstra HE, Coyne JA (2007) The locus of evolution: evo devo and the genetics of adaptation. Evolution 61: 995–1016. 17492956 10.1111/j.1558-5646.2007.00105.x

[57] Carroll SB (2008) Evo-Devo and an Expanding Evolutionary Synthesis: A Genetic Theory of Morphological Evolution. Cell 134: 25–36. 18614008 10.1016/j.cell.2008.06.030

[58] Li H, Durbin R (2009) Fast and accurate short read alignment with Burrows–Wheeler transform. Bioinformatics 25: 1754–1760. 19451168 10.1093/bioinformatics/btp324 PMC2705234

[59] Fiston-Lavier A-S, Singh ND, Lipatov M, Petrov DA (2010) Drosophila melanogaster recombination rate calculator. Gene 463: 18–20. 20452408 10.1016/j.gene.2010.04.015

