# Supplementary material for: Genomic Variation and Its Impact on Gene Expression in Drosophila melanogaster
Source: PLoS Genet. 2012 Nov 15;8(11):e1003055. doi: 10.1371/journal.pgen.1003055 (PMC3499359; doi:10.1371/journal.pgen.1003055)
Supplement: Figure S1 — Results of variant genotyping. Size (reference bases in thousands, left y-axis) of variants after Stage 2 (re-alignment, yellow) and Stage 3 (genotyping, blue), ordered by sequencing coverage (red line, right y-axis). This figure illustrates that the genotyping stage improves the consistency of variant calling materially, particularly for low-coverage genomes. (PDF) [file pgen.1003055.s001.pdf]

Supplementary Figures

(Please see Supplementary Figure Legends in Main Article Document)

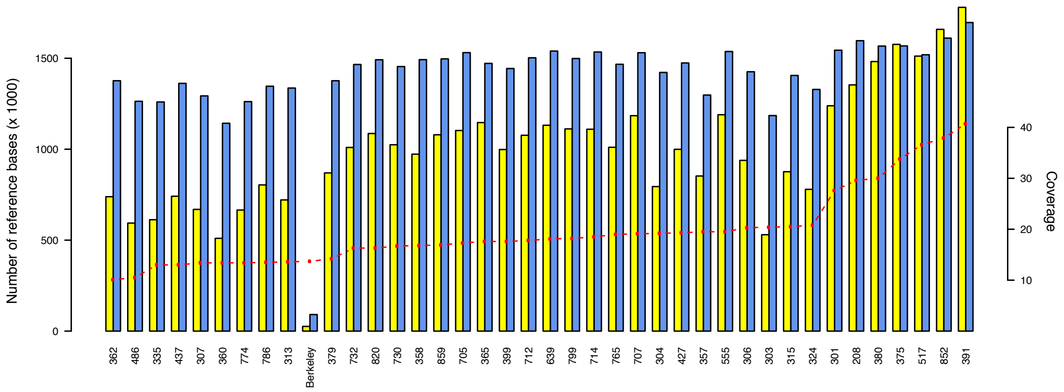

Figure S1.
