# Supplementary figures and images for: Genomic Variation and Its Impact on Gene Expression in Drosophila melanogaster
Source: PLoS Genet. 2012 Nov 15;8(11):e1003055. doi: 10.1371/journal.pgen.1003055 (PMC3499359; doi:10.1371/journal.pgen.1003055)

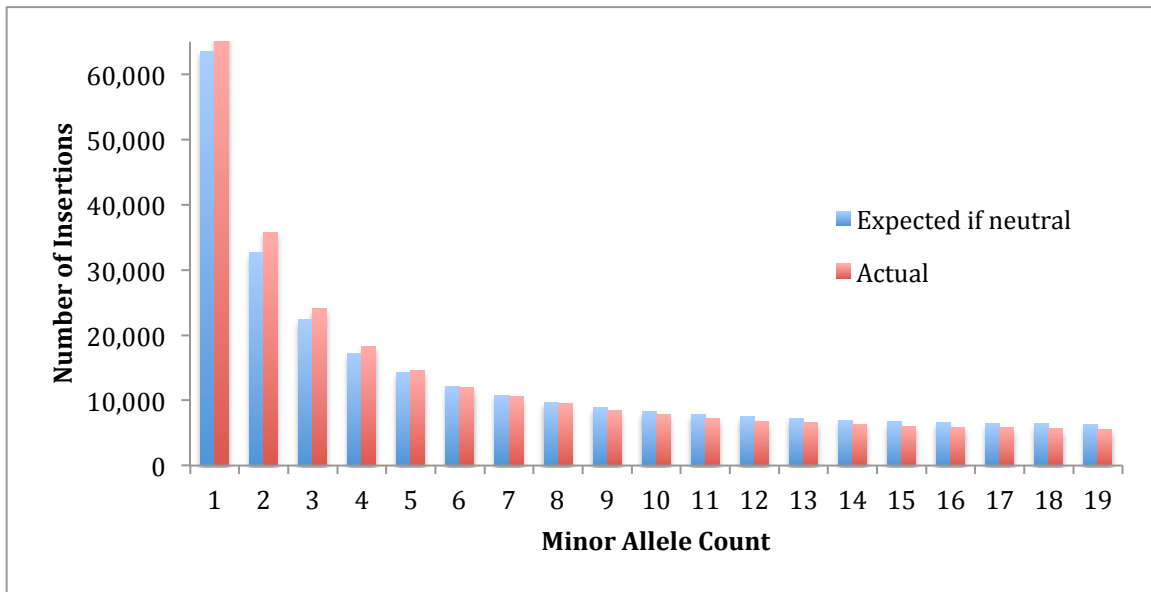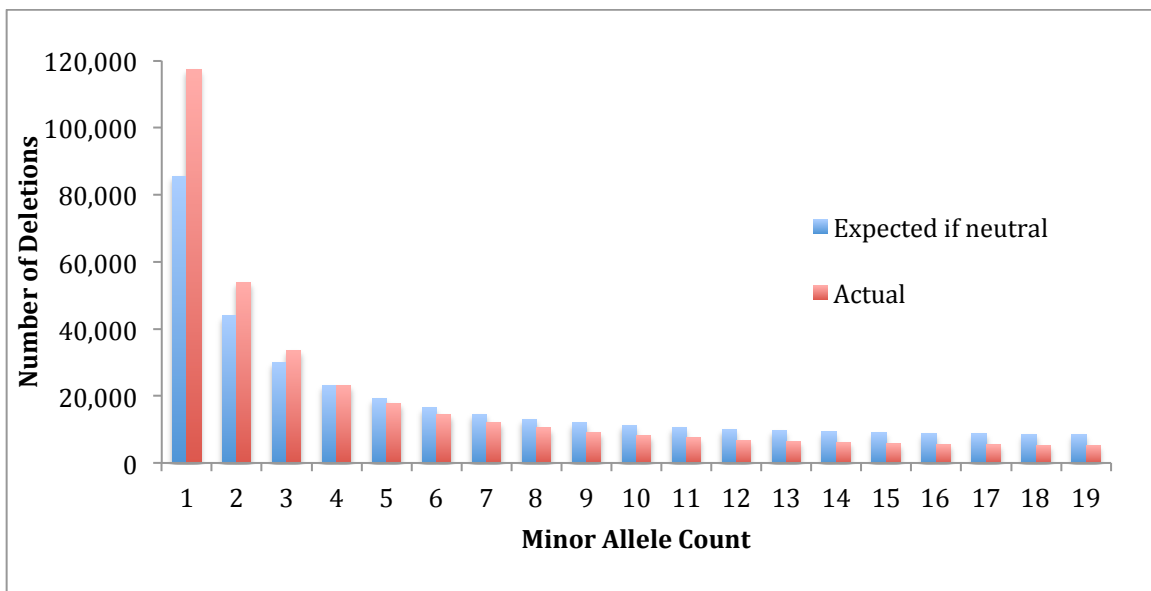

**Figure S2.** (first part)

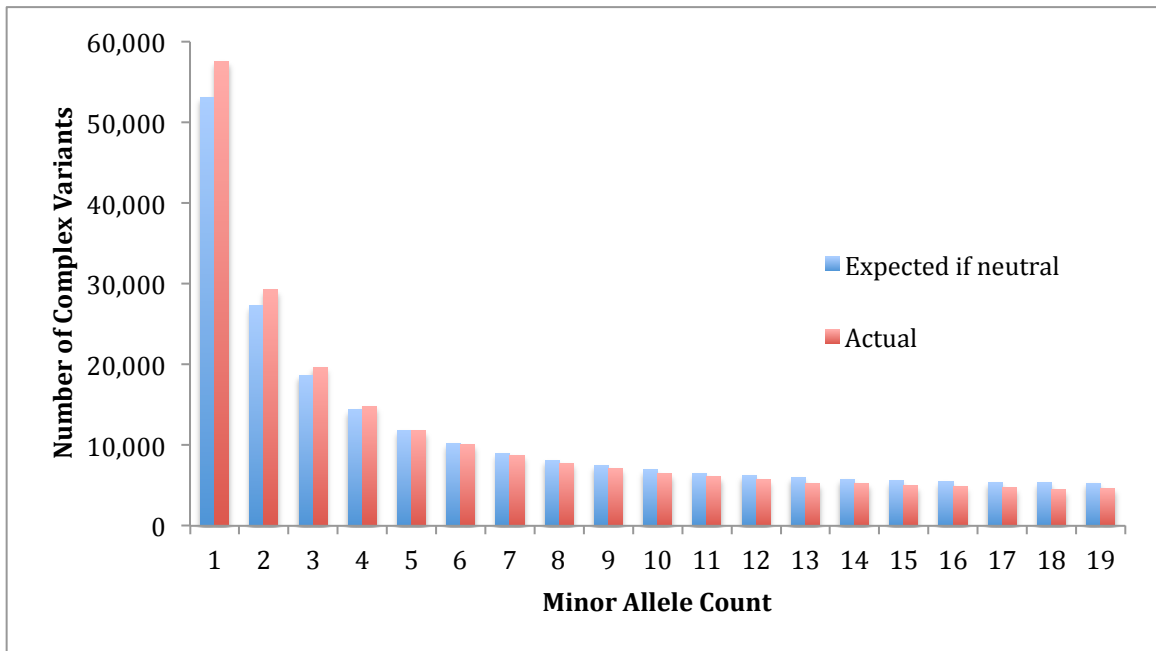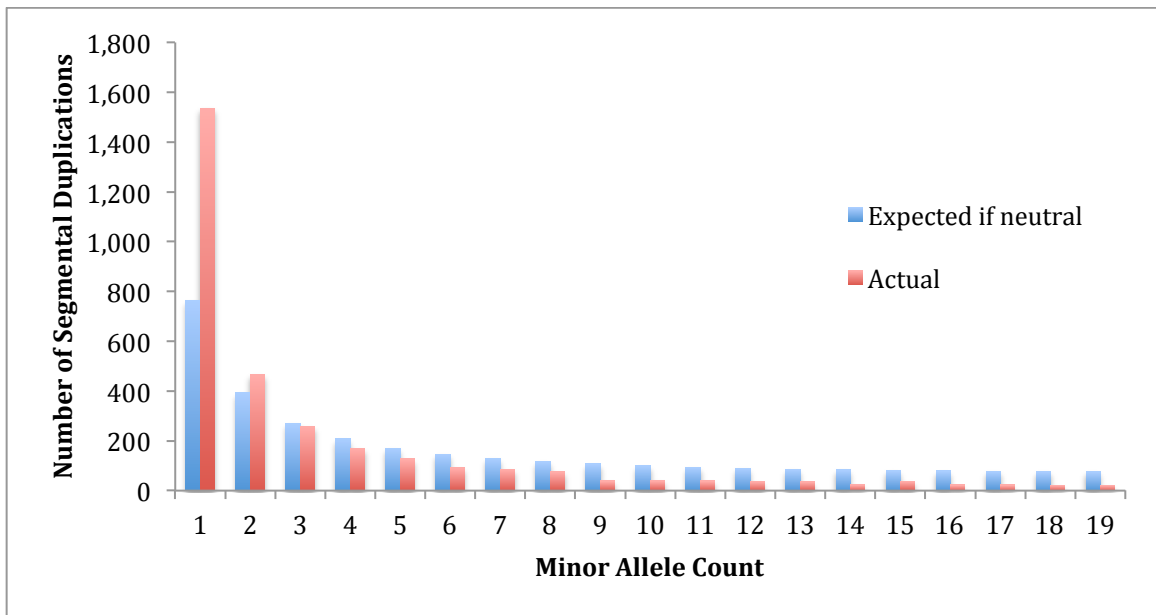

Figure S2. (Second part)

Supplement: Figure S2 — Number of variants by minor allele count. The number of insertions, deletions, complex variants and segmental duplications/copy number variants discovered is plotted next to the number expected under the neutral hypothesis. Deletions and especially segmental duplications are more enriched for low allele counts, which suggests that they are more under negative selection than the other variant types. (PDF) [file pgen.1003055.s002.pdf]

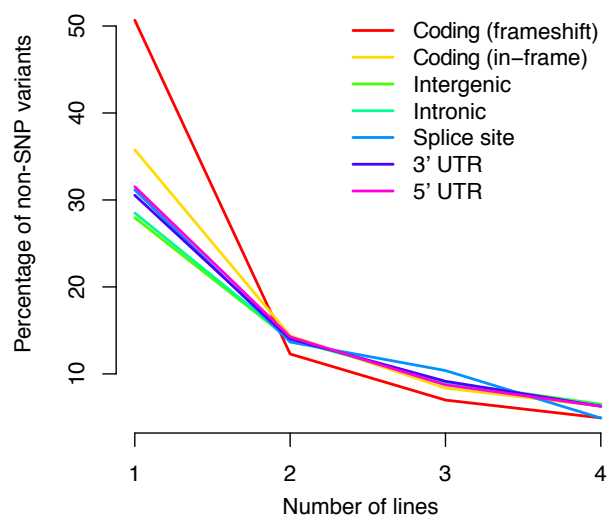

**Figure S3.**

Supplement: Figure S3 — Allele-frequency spectrum of non-SNP variants by genomic location. (PDF) [file pgen.1003055.s003.pdf]

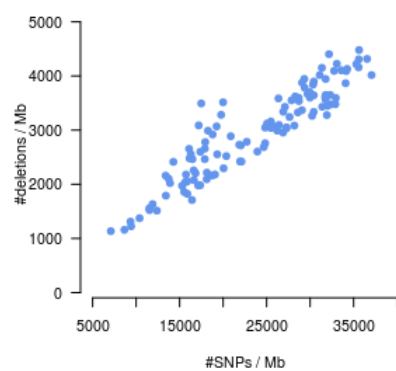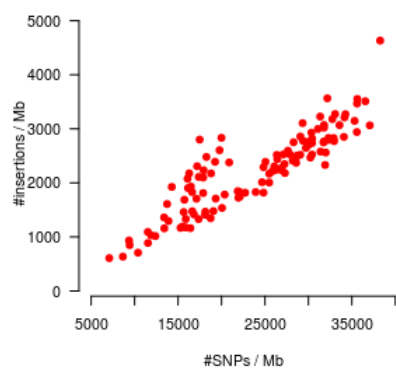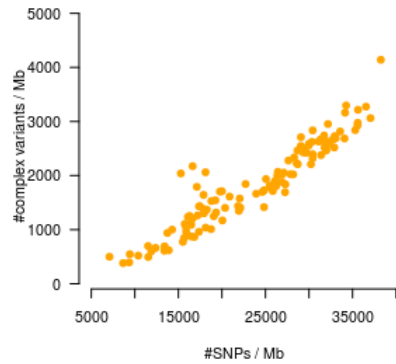

**Figure S5.**

Supplement: Figure S5 — Genome-wide correlation between SNP and non-SNP densities. The concentration (variants per kb) of SNPs is correlated with deletions (blue), insertions (red), and complex variants (orange). Densities were calculated in non-overlapping genomic bins of 50 kb across all autosomes and the X chromosome. (PDF) [file pgen.1003055.s005.pdf]

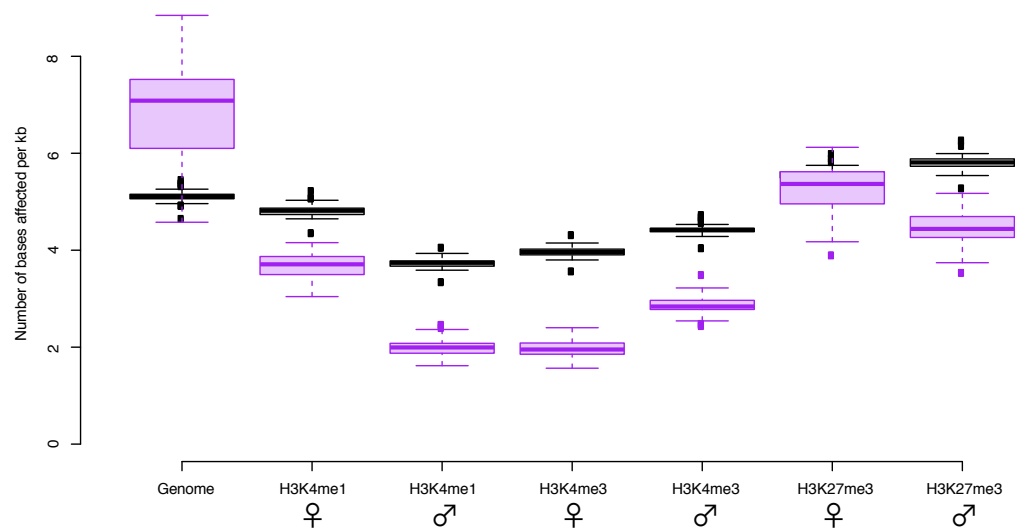

**Figure S6.**

Supplement: Figure S6 — Variant concentration in histone modified regions. The histone marks for adult flies were obtained from modENCODE. SNPs are in black/grey, indels and complex variants in purple. (PDF) [file pgen.1003055.s006.pdf]

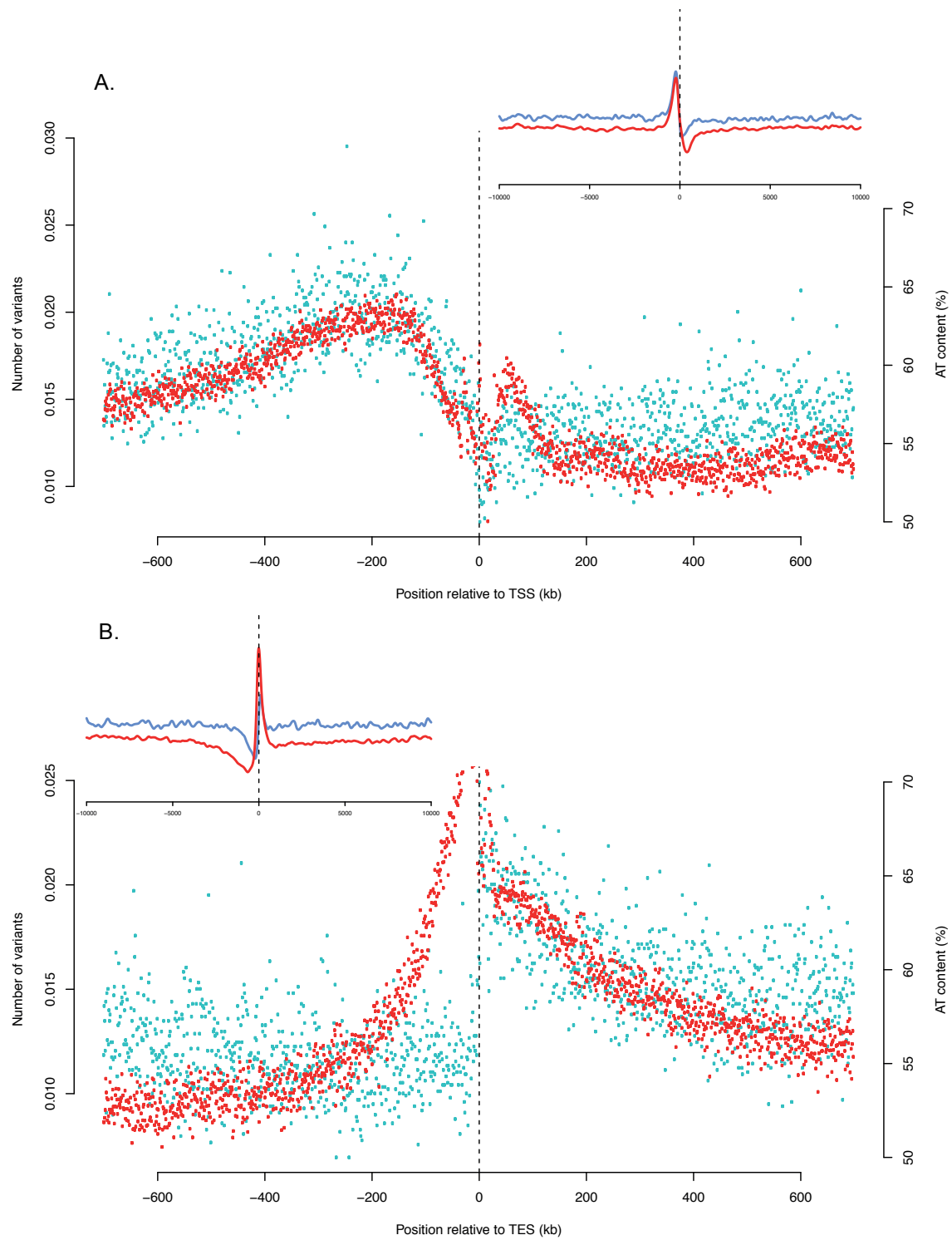

**Figure S7.**

Supplement: Figure S7 — Variant density and AT content (A) near TSS, (B) near TES. The inlet shows the same plot between 10 kb up- and downstream of respectively the TSS and TES (spline-smoothed). Blue dots depict variant density and red dots AT content (%). (PDF) [file pgen.1003055.s007.pdf]

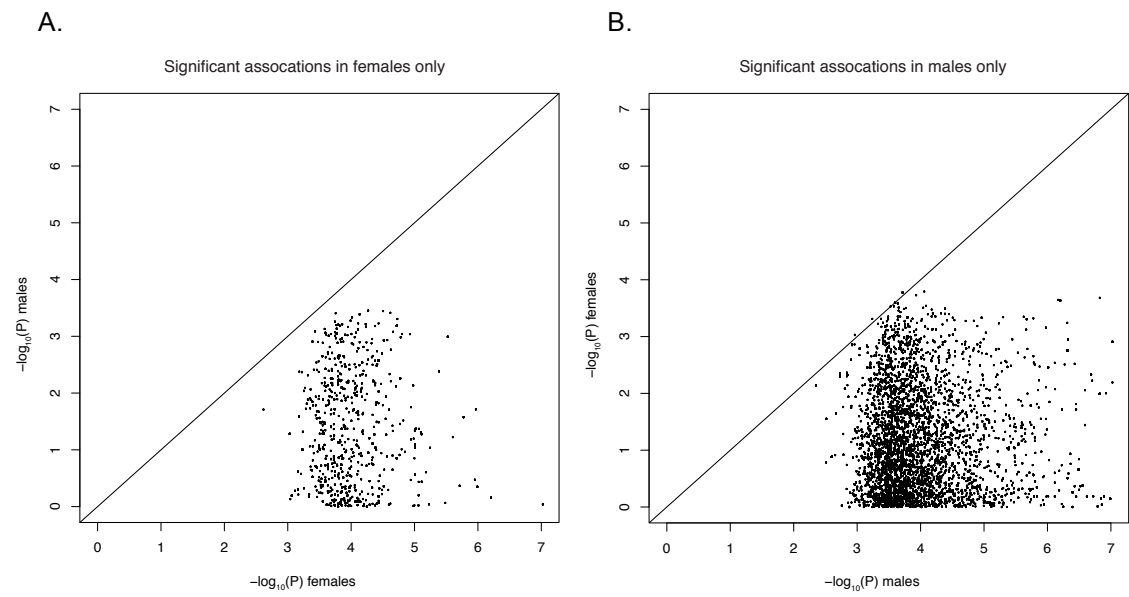

**Figure S8.**

Supplement: Figure S8 — Comparison of P-values for sex-specific associations. (a) Female-specific and (b) male-specific association P-values have mostly no significant counterpart for the other sex, ruling out the possibility that they labeled sex-specific only for marginally failing to meet the significance threshold. (PDF) [file pgen.1003055.s008.pdf]

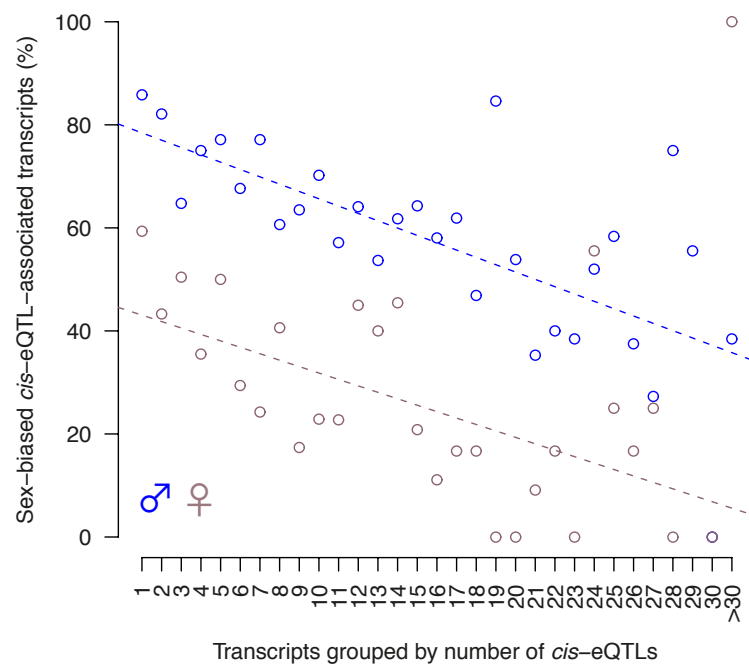

**Figure S9.**

Supplement: Figure S9 — Correlation between sex-bias and number of cis-associations. Transcripts were grouped by the number of cis-eQTLs and the y-axis indicates the percentage of transcripts for which cis-associations were detected in one sex only. Linear regression fits are plotted for both sexes separately. (PDF) [file pgen.1003055.s009.pdf]

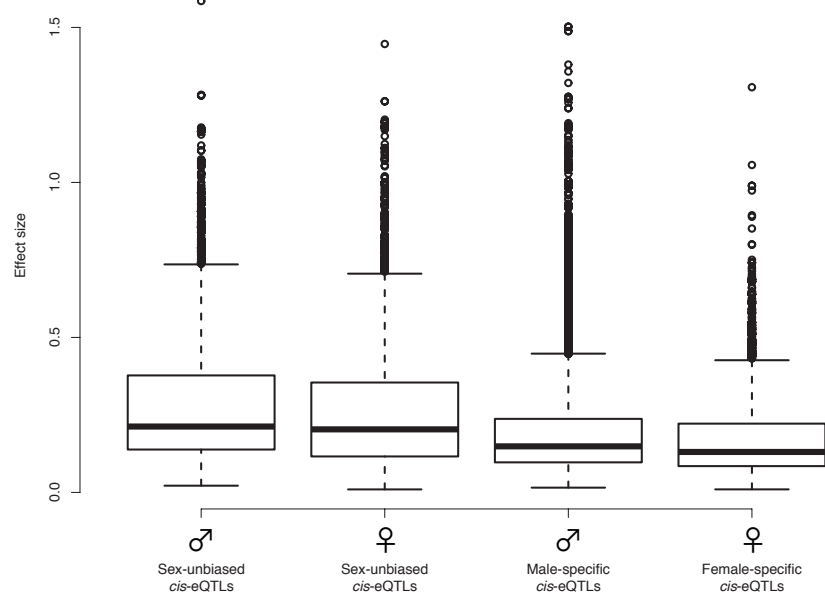

**Figure S10.**

Supplement: Figure S10 — Effect size of sex-biased and unbiased cis-eQTLs. (PDF) [file pgen.1003055.s010.pdf]

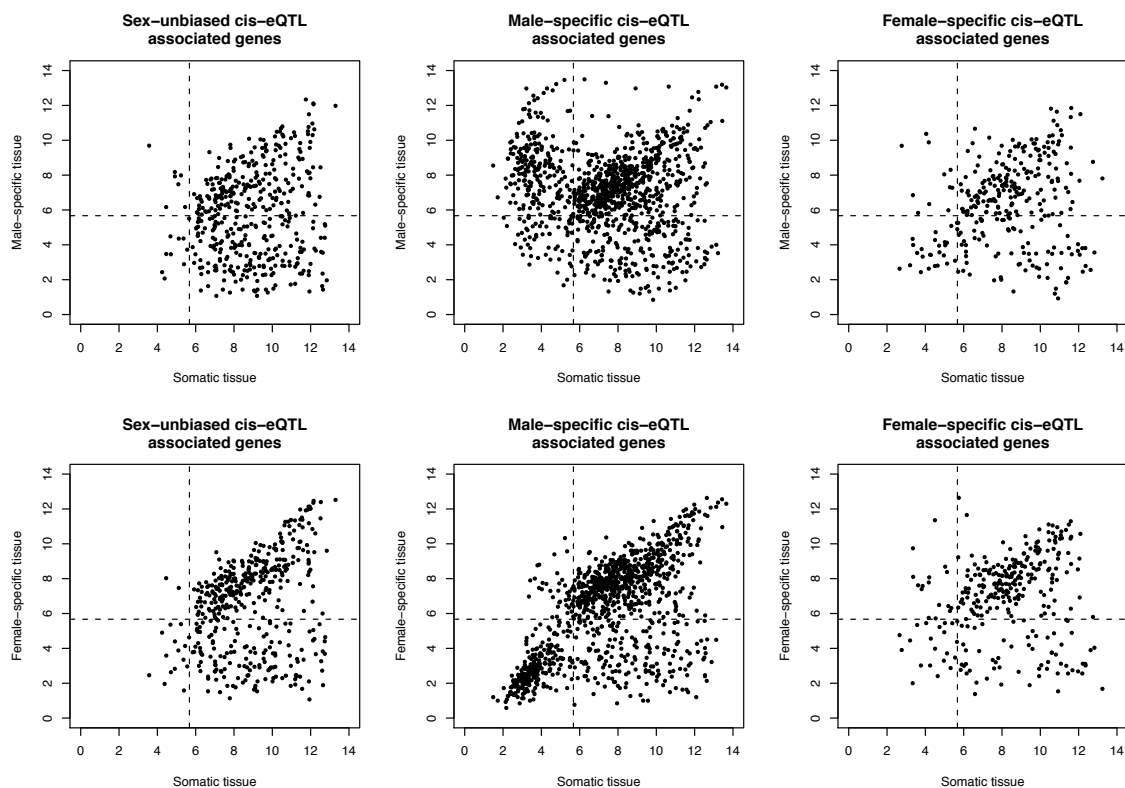

**Figure S11.**

Supplement: Figure S11 — Tissue-specific gene expression pattern analysis of cis-eQTL-associated genes. Scatter plots showing the highest expression (log2) of cis-eQTL-associated genes in 12 somatic tissues (X-axis) and either male-specific (i.e., testis or accessory gland; Y-axis, upper panels) or female-specific tissues (i.e., ovary or spermatheca; Y-axis, lower panels). Dashed horizontal and vertical lines denote the expression level at which we considered transcripts as expressed (see Methods for details). (PDF) [file pgen.1003055.s011.pdf]

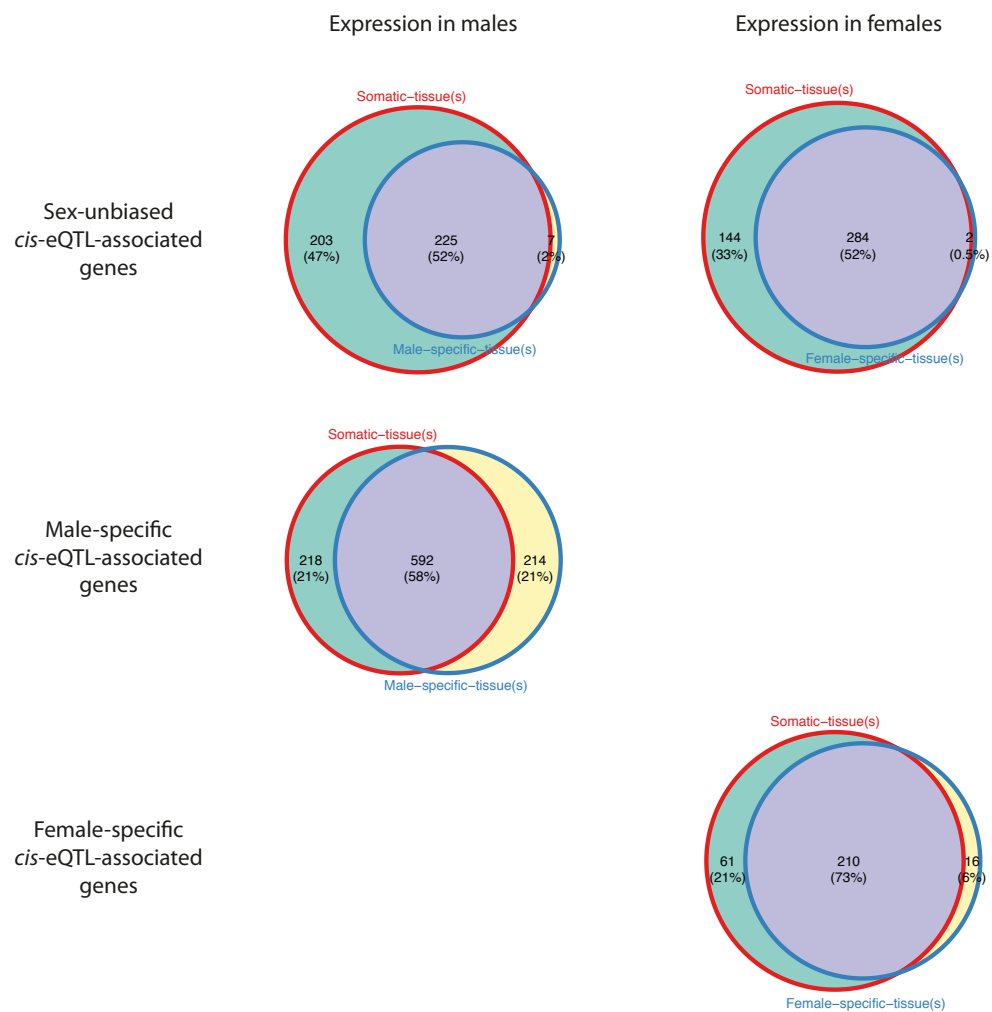

**Figure S12.**

Supplement: Figure S12 — Venn diagrams depicting the percentage of cis-eQTL-associated genes that are expressed either in somatic tissues only, sex-specific tissues only, or both. Genes not expressed in any tissue or for which tissue-specific gene expression data was missing were not considered in this analysis. (PDF) [file pgen.1003055.s012.pdf]

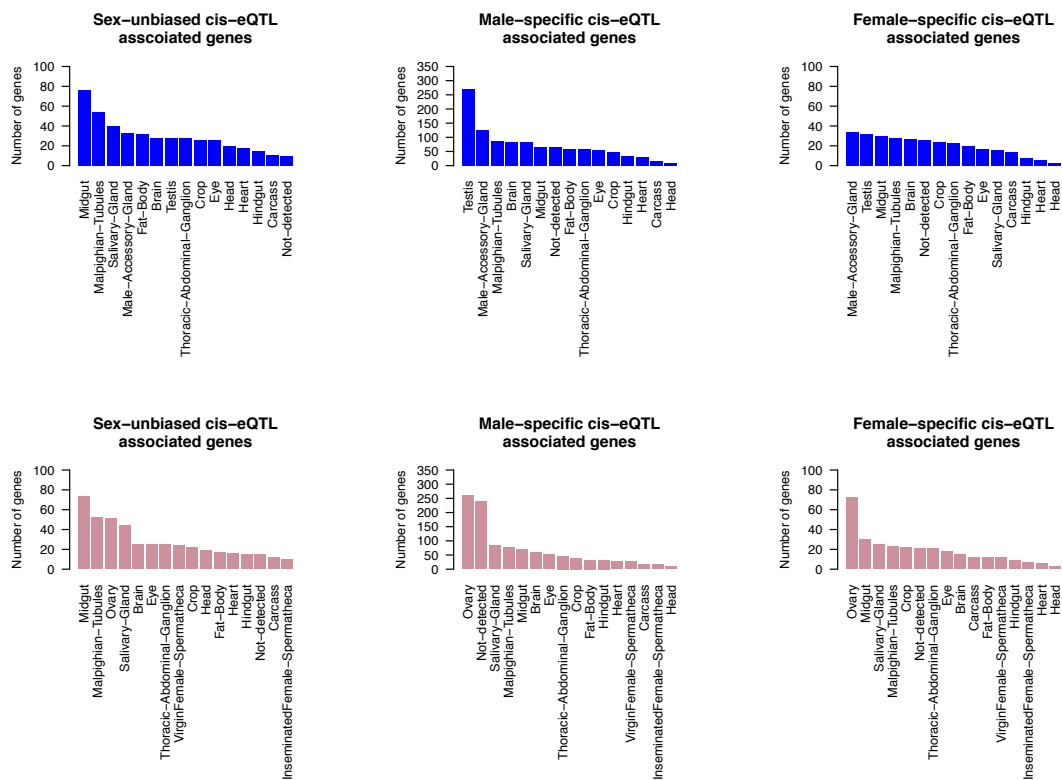

**Figure S13.**

Supplement: Figure S13 — Bar graphs showing the number of cis-eQTL-associated genes that exhibit highest expression in the respective tissues in either males (i.e., including testes and accessory gland; upper panels) or females (i.e., including ovary and spermatheca; lower panels). Genes classified as being not expressed in any tissue are denoted as ‘not-detected.’ (PDF) [file pgen.1003055.s013.pdf]

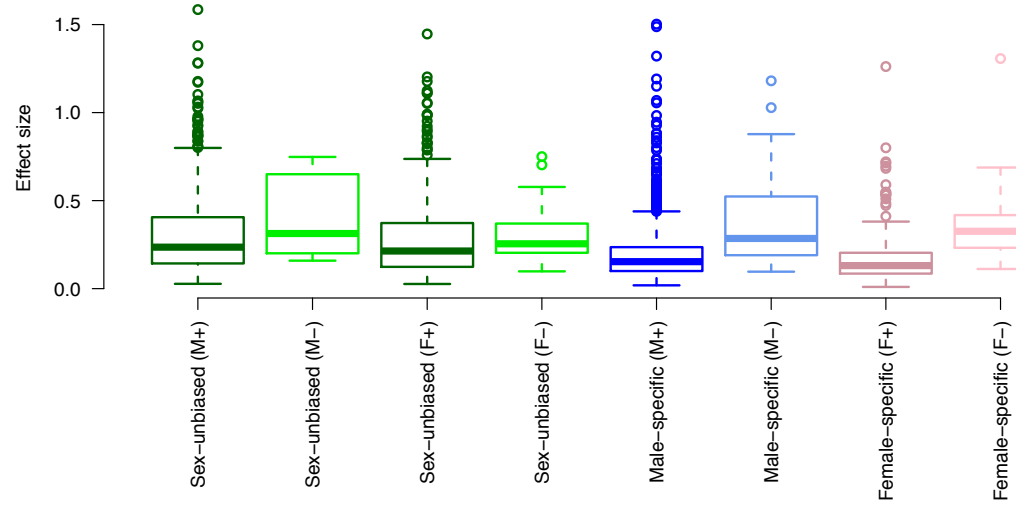

**Figure S14.**

Supplement: Figure S14 — cis-eQTL effect size by category (i.e., sex-unbiased, male-, or female-specific) and by their gene expression status among different tissues [45]. M+: expressed in males; M−: not expressed in males; F+: expressed in females; F−: not expressed in females. (PDF) [file pgen.1003055.s014.pdf]

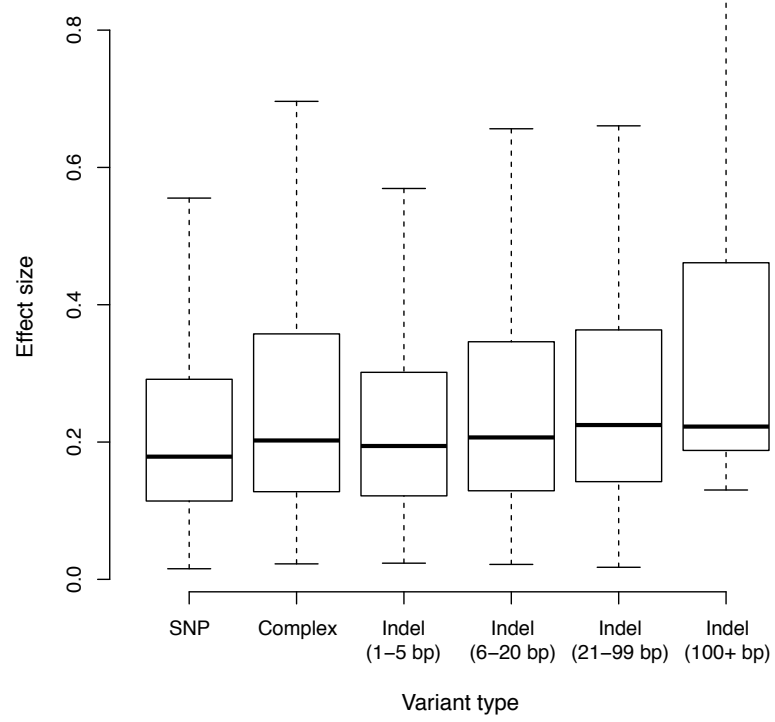

**Figure S15.**

Supplement: Figure S15 — Effect size of cis-eQTLs by variant type (i.e., SNP, indel, complex variant) and indel size. (PDF) [file pgen.1003055.s015.pdf]

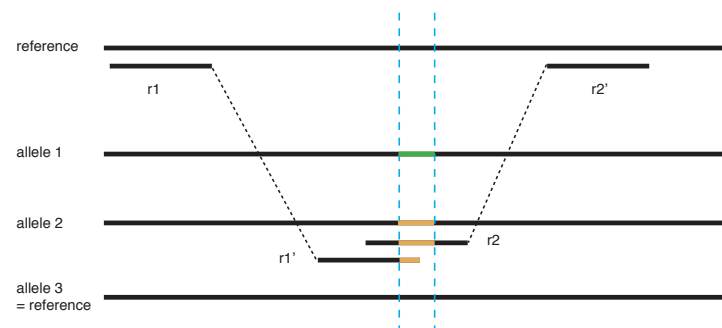

**Figure S16.**

Supplement: Figure S16 — Illustration of differential read pair alignment for variant imputation. In this example, there are three alleles discovered after the first two stages of variant calling in the population. Read pairs are aligned to all alleles and the reference sequence. Reads r1' and r2 best align to allele 2, i.e., they either only align to this allele or produce the lowest number of mismatches when aligned to allele 2 compared to the alternatives. Thus allele 2 receives two positive and zero negative votes, while the other two alleles receive zero positive and two negative votes. A new variant call is made when the positive exceed the negative votes by at least one. An existing variant call (i.e., a variant called in stages 1 and 2) is removed when the positive votes are not greater than half the negative votes. In all cases the votes are recorded as tags in the variant list. (PDF) [file pgen.1003055.s016.pdf]
