# Supplementary material for: Genomic Variation and Its Impact on Gene Expression in Drosophila melanogaster
Source: PLoS Genet. 2012 Nov 15;8(11):e1003055. doi: 10.1371/journal.pgen.1003055 (PMC3499359; doi:10.1371/journal.pgen.1003055)
Supplement: Figure S4 — Evolutionary conservation analysis of insertions in the Rh6 coding sequence. Both insertions were predicted in all 39 lines and are also present in seven out of eleven Drosophila species other than D. melanogaster. Moreover, the resulting gene model supports an Rh6 cDNA clone of the OregonR/white strain. Thus, the reference genome has a likely rare allele, which disrupts a splice site and introduces a premature stop codon resulting in a truncated protein. (PDF) [file pgen.1003055.s004.pdf]

```
 INS-3R:11309705      CCTACTGGCACATCATG
 INS-3R:11309707                         GG
 D. melanogaster  TTCT-----------------AA--TA
     D. simulans  TTCTCCTACTGGCACATCATGAAGGTA
    D. sechellia  TTCTCCTACTGGCACATCATGAAGGTA
       D. yakuba  TTCTCCTACTGGCACATCATGAAGGTA
       D. erecta  TTCTCCTACTGGCACATCATGAAGGTA
    D. ananassae  TTCTCCTACTGGCACATCATGAAGGTG
D. pseudoobscura  TTCTCCTACTGGCACATCATGAAGGTA
   D. persimilis  TTCTCCTACTGGCACATCATGAAGGTA
   D. willistoni  TTCTCCTA-------------------
      D. virilis  TTCTCATA-------------------
   D. mojavensis  TTCTCCTA-------------------
    D. grimshawi  TTCTCATA-------------------
```

Figure S4.
