# Supplementary material for: Genomic Variation and Its Impact on Gene Expression in Drosophila melanogaster
Source: PLoS Genet. 2012 Nov 15;8(11):e1003055. doi: 10.1371/journal.pgen.1003055 (PMC3499359; doi:10.1371/journal.pgen.1003055)
Supplement: Text S1 — Supplementary Information. (DOC) [file pgen.1003055.s029.doc]

**Supplementary Information**

**Genomic variation and its impact on gene expression in**

***Drosophila* *melanogaster***

Andreas Massouras^1*^, Sebastian M. Waszak^1*^, Monica Albarca-Aguilera^1^, Korneel Hens^1^, Wiebke Holcombe^1^, Julien F. Ayroles^2^, Emmanouil T. Dermitzakis^3^, Eric A. Stone^4^, Jeffrey D. Jensen^5^, Trudy F.C. Mackay^4^, and Bart Deplancke^1†^

^*^These authors contributed equally to this work

†To whom correspondence should be addressed. E-mail: [bart.deplancke@epfl.ch](mailto:bart.deplancke@epfl.ch) (B.D.)

**This file contains:**

- Supplementary Information
- Supplementary References

**Supplementary Information**

**Whole genome re-alignment**

We used MUMer 3.22 to obtain an initial list of maximum unique matches (MUMs) between chromosomes (minimum length of 20 bp). We sorted this list with the longest increasing subsequence (LIS) algorithm and removed overlapping MUMs. We further filtered out MUMs that were shorter than 10 kb to reduce the total number of gaps. Iterative gap closing between two consecutive MUMs was performed with the memory-efficient global alignment tool stretcher (EMBOSS) . In each iteration step, MUMs were part of the sub-alignment and served as anchors to ensure correctly resolved variant breakpoints. After each iteration step, we extracted variants from the global alignment. In rare cases, MUMs were part of the variant (*e.g.,* due to tandem repeats) and we used a backtracking strategy to resolve this erratic behavior, *i.e.* we re-used the previous MUM as a ‘left’ anchor and iteratively skipped ‘right’ MUMs until we obtained a MUM that was not part of the resulting variant.

**Genotyping**

We used a global variant list for all lines as discovered in stages 1 and 2 (**Online Methods**). The genotyping tool works on a single line at a time, using the whole genome sequencing reads (mostly paired-end) for that line and drawing variants from the global list. Reads are aligned to both the reference genome and all stage 2 target genomes (each containing the variant calls predicted for one line). It discards pairs of reads aligning to more than one part of the reference. Those reads aligning to one site at the reference are only allowed to align to the same site in all stage 2 genomes. Those reads not aligning at all to the reference, are discarded if they align to more than one different site at the stage 2 genomes. Since the stage 2 genomes already contain many variant calls, the alignment criteria are more stringent, allowing fewer mismatches. When a read pair is aligned to a particular site, only the alignment to the genome that produces the fewest mismatches is considered. The variants in that genome overlapping with the read are then given a “positive vote”, with all other alleles at the same site given a “negative vote”. In this way, reads best aligning to a stretch with no variants will give negative votes to all variants at that site (**Figure S16**). At the vote counting stage, variants that receive more positive than negative votes and are not already called for the line are added. Variants already called but whose positive votes are fewer than half of the negative votes are removed. Following this, a modified genome containing the now expanded set of variants is validated using the methodology employed with PrInSeS-G, namely by realigning reads using an independent tool (BWA) and then comparing the read depth at both breakpoints of each variant; variants that don’t contribute to an improved alignment are discarded.

We have been particularly careful to keep the false positive rate of our calls as low as possible. In particular, genotyping is prone to make false positive calls for repeated regions by selecting variants which result in near repeats becoming exact repeats. This is avoided by excluding alignment to multiple sites for different genomes. Further, the validation of read depth at each breakpoint filters out many false positive calls.

**Comparison between our variant dataset and the one reported by Emerson *et al.***

Emerson *et al*. report 1,392 deleted and 2,016 duplicated regions when mapped to version 5 of the *Drosophila* genome. We call 243 deletions of 100 bps or more (total 85,467 bps) that overlap with that dataset (829,810). Of these 243 deletions, 150 are identified in our dataset as segmental duplications. In addition, 14 of our insertion calls of 100 bps or more map with 90% similarity to the duplicated regions (total size 2,415,965 bps) reported by Emerson *et al*. . The relatively small overlap between variants called by the two studies may have several causes. For example, the study by Emerson *et al.* used array-CGH technology to call variants and as a result, the coordinates they report are imprecise (as compared to the nucleotide resolution offered in our study). The study also used *D. melanogaster* lines that are not part of the DGRP resource. Moreover, both Emerson *et al.*  and Cridland and Thornton present evidence that most CNPs are under strong purifying selection and as such constitute rare alleles (i.e. individual-specific); our segmental duplication calls also agree, being strongly enriched for very low allele counts (**Figure S2**). Finally, the release of version 5 of the *Drosophila* genome, that we use, may have already incorporated a different copy number for some regions than version 4, which Emerson *et al*. used.

**Supplementary References**

1. Kurtz S, Phillippy A, Delcher AL, Smoot M, Shumway M, et al. (2004) Versatile and open software for comparing large genomes. Genome Biology 5: R12.

2. Rice P, Longden I, Bleasby A (2000) EMBOSS: the European Molecular Biology Open Software Suite. Trends Genet 16: 276 - 277.

3. Emerson JJ, Cardoso-Moreira M, Borevitz JO, Long M (2008) Natural Selection Shapes Genome-Wide Patterns of Copy-Number Polymorphism in Drosophila melanogaster. Science 320: 1629-1631.

4. Cridland JM, Thornton KR (2010) Validation of rearrangement break points identified by paired-end sequencing in natural populations of Drosophila melanogaster. Genome Biol Evol 2: 83-101.
